# AMPKα1 Deficiency in Macrophages Impairs Tendon Regeneration and Tendon Stem Cell Function via TNF-α-FBP2 Signaling

**DOI:** 10.7150/ijbs.125245

**Published:** 2026-01-22

**Authors:** Lisha Zhu, Yu Wang, Xinmeng Shi, Min Yu, Xinjia Cai, Liyuan Chen, He Zhang, Xiaolan Wu, Chengye Ding, Hangbo Liu, Shiying Zhang, Chang Li, Tianhao Wu, Nan Jiang, Yan Liu

**Affiliations:** 1 Central Laboratory, Peking University School and Hospital of Stomatology, Beijing 100081, China.; 2 Department of Orthodontics, Peking University School and Hospital of Stomatology, Beijing 100081, China.; 3 National Center for Stomatology & National Clinical Research Center for Oral Diseases & National Engineering Laboratory for Digital and Material Technology of Stomatology & Beijing Key Laboratory of Digital Stomatology & Research Center of Engineering and Technology for Computerized Dentistry Ministry of Health & NMPA Key Laboratory for Dental Materials, Beijing 100081, China.; 4 Shanghai Engineering Research Center of Tooth Restoration and Regeneration & Tongji Research Institute of Stomatology & Department of Orthodontics, Shanghai Tongji Stomatological Hospital and Dental School, Tongji University, Shanghai 200072, China.; 5 Beijing Advanced Center of Cellular Homeostasis and Aging-Related Diseases, Institute of Advanced Clinical Medicine, Peking University, Beijing 100191, China.

**Keywords:** macrophage, adenosine monophosphate-activated protein kinase, fructose-1,6-bisphosphatase 2, tendon stem/progenitor cells, tendon regeneration

## Abstract

Tendon healing is limited by the minimal intrinsic regenerative capacity of the tissue, resulting in the formation of fibrovascular scar tissue rather than functional regeneration. Macrophage immunometabolism governs the balance between inflammation and repair; however, its effects on tendon regeneration are poorly understood. In this study, we investigated the differential activation of macrophage AMP-activated protein kinase (AMPK) and its phenotypic alterations in neonatal and adult tendon injury models. Using myeloid-specific AMPKα1 knockout (*LysM-Cre; Ampkα1^fl/fl^*) mice, we found that macrophage AMPKα1 deficiency impairs tendon regeneration and repair capacity, leading to compromised proliferation, migration, and differentiation functions of tendon stem/progenitor cells (TSPCs). Mechanistically, AMPKα1-deficient macrophages exhibited increased TNF-α production, which promoted the expression of Fructose-bisphosphatase 2 (FBP2) in a PI3K/AKT-dependent manner. In addition, FBP2 can modulate mitochondrial biogenesis and dynamics through a non-enzymatic mechanism and facilitate tissue repair and regeneration. Collectively, these findings underscore the immunometabolism mechanism linking macrophage AMPKα1 activity to stem cell injury responses via a TNF-α-FBP2 axis and provide new insights into the role of FBP2 for regulating stem cells.

## Introduction

Tendon injury is a challenging and prevalent motor system disease that compromises daily activities and leads to disability 1. Failure of the injury repair process is often attributed to insufficient regenerative capacity and dysregulation of the immune microenvironment 1, 2. Macrophages, as pivotal components of the innate immune system, play versatile roles in maintaining tissue homeostasis 3-5. Initially, they exhibit a pro-inflammatory phenotype but subsequently repolarize toward an anti-inflammatory phenotype to actively support tendon regeneration 6, 7. In recent years, several studies have investigated the influence of macrophage behavior on tendon regeneration 8, 9. However, the molecular mechanisms governing macrophage function in the tendons remain largely unknown.

Effective tendon regeneration requires coordinated interactions between immune cells and tendon stem/progenitor cells (TSPCs). Macrophages secrete a range of immune cell mediators that regulate stem cell proliferation and govern stem cell-mediated tissue repair 10-12. TSPCs play a crucial role in tendon repair and are characterized by their robust tenogenic and self-renewal potential 13. They are highly enriched, maintained by their microenvironment (the 'niche'), and respond robustly to environmental stimuli 14. However, the mechanism by which macrophages affect stem cell behavior remains unclear. Our previous work revealed that the regenerative capacity of tendons in the neonatal period was superior to that in adults 15, and emerging literature has demonstrated that macrophages of different ages have distinct effects on tissue repair 16, 17. For example, neonatal macrophages exhibit weak inflammatory responses and effectively enhance cell proliferation 18. Therefore, elucidating the molecular mechanisms underlying this differential response pattern is critical for modulating macrophage function and could provide potential regulatory targets for tendon regeneration.

AMP-activated protein kinase (AMPK) regulates multiple metabolic processes that maintain cellular energy homeostasis 19. Activation of this pathway is essential for metabolic and functional adaptations of macrophages 20. Specifically, phosphorylation of AMPKα1 contributes to the switch in the macrophage phenotype from pro- to anti-inflammatory 21, 22. However, whether macrophage AMPKα1 plays a functional role in tendon repair remains unknown. In this study, we demonstrated the functional disparity and differential activation of AMPKα1 in macrophages following tendon injury in both newborn and adult mice. Using a conditional genetic knockout mouse model, we investigated not only the influence of AMPKα1 deficiency in macrophages on tendon repair quality but also its mechanistic effects on TSPCs. Our results demonstrate that fructose-bisphosphatase 2 (FBP2), a multifunctional protein in the downstream TNF-α/AKT signaling 23, 24, impairs the function and regenerative capacity of TSPCs by compromising mitochondrial integrity and function. Overall, we identified a previously unknown role of macrophage AMPKα1 in regulating tendon tissue repair and revealed a key biological mechanism of TNF-α/AKT/FBP2 in response to mitochondria damage and tendon injury. These findings provide new insights into the enhancement of endogenous regenerative capacity.

## Materials and Methods

### Animals

Male C57 mice aged 0-1 day and 12 weeks and 6-8-week-old BALB/c nude mice were purchased from Beijing Vital River Laboratory Animal Technology (China). *LysM-Cre* mice were crossed with *Ampkα1^flox/flox^* mice to generate transgenic mice with specific deletion of macrophage *Ampkα1* through backcrossing and subsequent progeny selection (*LysM-Cre*;* Ampkα1^fl/fl^* mice), along with littermate controls (*Ampkα1^fl/fl^*). Genomic DNA from the mouse tail was used for genotype identification by polymerase chain reaction (PCR). The DNA primers are listed in Table S1. All animals were housed in ventilated cages under a 12/12-h light/dark cycle with ad libitum access to food and water. During different experiments, mice were excluded if they died prematurely or suffered from tumors.

### Tendon transection models

For tendon transection, mice were intraperitoneally injected with 1% pentobarbital (0.1 mL per 100 g). After complete anesthesia, the Achilles tendon was exposed through lateral transection. A transverse incision of the Achilles tendon was made on each leg using microsurgical instruments. The wound was irrigated, and the skin was closed with sutures. Subsequently, the mice were euthanized at planned postoperative time points (1, 4, and 8 weeks) with an overdose of anesthesia, and the repair tissues were collected. Specifically, the wild-type mice (including neonatal and adult mice) were used to study the acute phase of inflammation and early regeneration, which typically peaks within 7 days. In contrast, the AMPKα1-deficient model was designed to assess long-term regeneration and tissue maturation, which require longer time points (4-8 weeks).

### *In vivo* knockdown of Fbp2 by AAV9-shRNA

For tendon-specific knockdown of *Fbp2* in mice, AAV9 vectors expressing shRNA targeting *Fbp2* (AAV9-shRNA-*Fbp2*) or a non-targeting control shRNA (AAV9-shRNA-NC, Ctrl AAV) were constructed by GenePharma (Shanghai, China). Neonatal mice received a local injection of 10 µL of AAV9-shRNA-*Fbp2* or AAV9-shRNA-NC at a titer of 1 × 10¹² viral genomes (vg) /mL. Adult mice were administered 30 µL of the same viral preparation via local injection. Tendon tissues were harvested at 4 and 8 weeks post-injection. The targeting sequence for AAV9-shRNA-*Fbp2* was 5'-CTGGATGGATCTTCAAACATT-3'; the control shRNA sequence was 5'-TTCTCCGAACGTGTCACGT-3'.

### Isolation and culture of TSPCs

Primary TSPCs were harvested from control (*Ampkα1^fl/fl^*) mice and *LysM-Cre*; *Ampkα1^fl/fl^* mice according to the established procedure 25, 26. The harvested tendons were minced and digested completely with 3 mg/mL collagenase type I (Thermo Fisher Scientific) and 4 mg/mL dispase (Roche) at 37°C for 1 h. After passing through a 70-μm strainer, single-cell suspensions were cultured in low-glucose Dulbecco's modified Eagle's medium (DMEM, Hyclone) supplemented with 15% fetal bovine serum (FBS; Gibco), 2 mM L-glutamine (Thermo Fisher Scientific), and 100 U/mL penicillin/streptomycin (Thermo Fisher Scientific) in an incubator at 37 °C with 5% CO_2_. When the cells reached 80-90% confluence, they were passaged. TSPCs at passages 1-3 were used for subsequent experiments. A list of reagents and resources is provided in Table S2.

### Isolation, culture, and polarization of bone marrow-derived macrophages (BMDMs)

BMDMs were isolated from control and *LysM-Cre*; *Ampkα1^fl/fl^* mice as previously described 25. Briefly, bone marrow cells were flushed from the femurs, isolated using Lymphoprep (STEMCELL), and cultured for 5-7 days in RPMI 1640 medium (Solarbio) containing 10% FBS (Gibco), 1% penicillin/streptomycin (Thermo Fisher Scientific), and 20 ng/mL recombinant mouse macrophage colony-stimulating factor (M-CSF, Peprotech). The culture medium was changed once every 3 days. After 5-7 days, the supernatant of macrophages from control and *LysM-Cre*; *Ampkα1^fl/fl^* mice were collected for further experiments. Macrophage polarization *in vitro* was performed as previously described 27.

### TSPC differentiation in BMDM-conditioned medium

For the differentiation experiments, TSPCs were cultured in 12-well plates (50,000 cells/well). Tenogenic differentiation was induced using a corresponding differentiation medium supplemented with the supernatant collected from the BMDM-conditioned medium at a ratio of 1:1. The tenogenic differentiation medium contained growth medium supplemented with 10 ng/mL TGF-β1 (Peprotech), 10 ng/mL GDF-5 (R&D System), and 0.05 mM l-ascorbic acid 2-phosphate. After 7 days of culture, quantitative real-time PCR (qRT-PCR) was performed to analyze the expression of tenogenic-related genes. Two weeks after tenogenic induction, the cells were assayed by Sirius Red staining (Solarbio, Beijing, China) to assess their tenogenic differentiation capacity.

### Small interfering RNA (siRNA) knockdown

siRNA sequences of the target gene markers were synthesized by GenePharma (China). Cells were diluted to a suitable density and seeded onto a plate. After 24 h at 70-80% confluence, the cells were treated with siRNA (50 nM) using the jetPRIME reagent (Polyplus, France) according to the manufacturer's protocol. The siRNA sequences are listed in Table S3.

### RT-qPCR

Total RNA was isolated from primary cells and tendon tissues using TRIzol reagent (Thermo Fisher Scientific) according to the manufacturer's instructions. Then, the mRNA was converted to complementary DNA by reverse transcription, and PCR was performed using gene-specific primers and SYBR Green (Thermo Fisher Scientific) on a 7900HT Fast Time PCR system. The synthesized primers are listed in Table S4.

### Western blotting

Total proteins from cell lysates and tissue homogenates were harvested using RIPA Buffer (Thermo Fisher Scientific) with a Protease/Phosphatase Inhibitor Cocktail (Thermo Fisher Scientific). Cell lysate proteins were separated using 10% SDS-polyacrylamide gel electrophoresis, transferred to polyvinylidene difluoride membranes, and blocked with 5% nonfat milk. The membranes were probed with corresponding primary antibodies overnight at 4 °C. The membranes were washed three times in TBS with 0.1% Tween-20 for 5-10 min each, incubated with the appropriate secondary antibodies for 1 h, washed twice in TBS with 0.1% Tween-20, and imaged. Detailed information on the primary and secondary antibodies is provided in Table S5.

### Staining of cells and tissue sections

Hematoxylin and eosin (HE), Sirius red, and Masson's trichrome staining were performed according to standard procedures to examine the general appearance of the soft tissues or cells. For immunofluorescence staining, sections (paraffin-embedded or frozen sections) or cells were incubated with anti-p-AMPK, anti-AMPK, anti-F4/80, anti-CD206, anti-NOS2, anti-CD86, anti-CD68, anti-Ki67, anti-SCX, anti-TNC, anti-TNMD, anti-FMOD, or anti-FBP2 antibodies. Next, sections or cells were incubated with fluorescein isothiocyanate (FITC) or rhodamine-conjugated secondary antibodies. Nuclei were counterstained with DAPI. Confocal microscopic images were acquired using a laser-scanning microscope (LSM 510, Zeiss, Germany) and processed using LSM 5 Release 4.2 software.

### Gene expression analysis

Differential gene expression was evaluated by transcriptome sequencing (RNA-Seq). tendon tissues from control and *LysM*-Cre; *Ampkα1^fl/fl^* mice (3 mice per condition, neonatal mice) were snap frozen in liquid nitrogen for further analyses. Subsequently, cDNA library construction, library purification, and transcriptome sequencing were performed according to the instructions of the Wuhan Huada Sequencing Company (www.genomics.org.cn; BGI, Shenzhen, China).

### Scanning electron microscope (SEM)

To characterize the fiber morphology of the neo-tissues using field emission scanning electron microscopy, all glass slide samples underwent dewaxing, dehydration, and subsequent gold sputter coating at 20 mA for 100 s. Thereafter, the microstructures of the neo-tissues were observed, and images were captured using SEM (Hitachi SU8020, Japan) at 15 kV.

### Transmission electron microscopy (TEM)

The ultra structures of the neo-tissues and cells were examined by TEM (Hitachi HT7700, Japan) at 100 kV. The samples were double-fixed with 1% osmium tetroxide (Sigma Aldrich), stained with lead citrate and uranyl acetate, and embedded in epoxy resin. Transverse and longitudinal ultrathin sections (70-100 nm) were prepared using a Leica ultramicrotome and placed on copper grids. Counting and measurement of the collagen diameter and mitochondrial mass from TEM images were performed using ImageJ software.

### Atomic force microscope (AFM)

The Young's modulus of tendon tissues was quantified using an atomic force microscope (AFM; Dimension Icon, Bruker, USA) in PeakForce Tapping mode. Scanning was performed at a rate of 1.0 Hz with an amplitude set point of 250 mV. A TAP150A probe was employed, and deflection sensitivity was calibrated against a sapphire reference sample. Data processing and analysis were conducted using Nanoscope Analysis software (version 1.60).

### Mitochondrial morphology and mass

The mitochondrial morphology and mass of TSPCs were evaluated by confocal immunofluorescence microscopy using the fluorescent dye MitoTracker Red (Beyotime, China) and by TEM analysis. In addition, mtDNA was quantified using qPCR to measure the ratio of mtDNA to genomic DNA. The primer sequences are presented in Table S4.

### Mitochondria electric potential (Δψ)

The mitochondrial membrane potential was monitored using JC1 staining (Solarbio, China). Briefly, cells were incubated with 10 μg/mL of JC-1 working solution for 20 min at 37°C in the dark. JC-1 monomer fluorescence (green) was detected at 525 nm (emission) and JC-1 polymer fluorescence (red) was detected at 590 nm (emission). The images were captured using a fluorescence microscope.

### Statistical analysis

The quantitative data are presented as means ± SD. Unpaired t-tests were performed to assess whether statistical differences existed between the two groups. Multiple comparisons were performed using one-way analysis of variance (ANOVA) and Tukey's post-hoc test, with *p*-values < 0.05 considered significant. The significance level is presented as *p < 0.05, ** *p* < 0.01, *** *p* < 0.001 and **** *p* < 0.0001. Statistical analyses were performed using GraphPad Prism® software (version 9.0, GraphPad Software Inc, California, USA).

## Results

### Activation of AMPKα1 in macrophages promotes neonatal tendon healing

**Figure 1:**
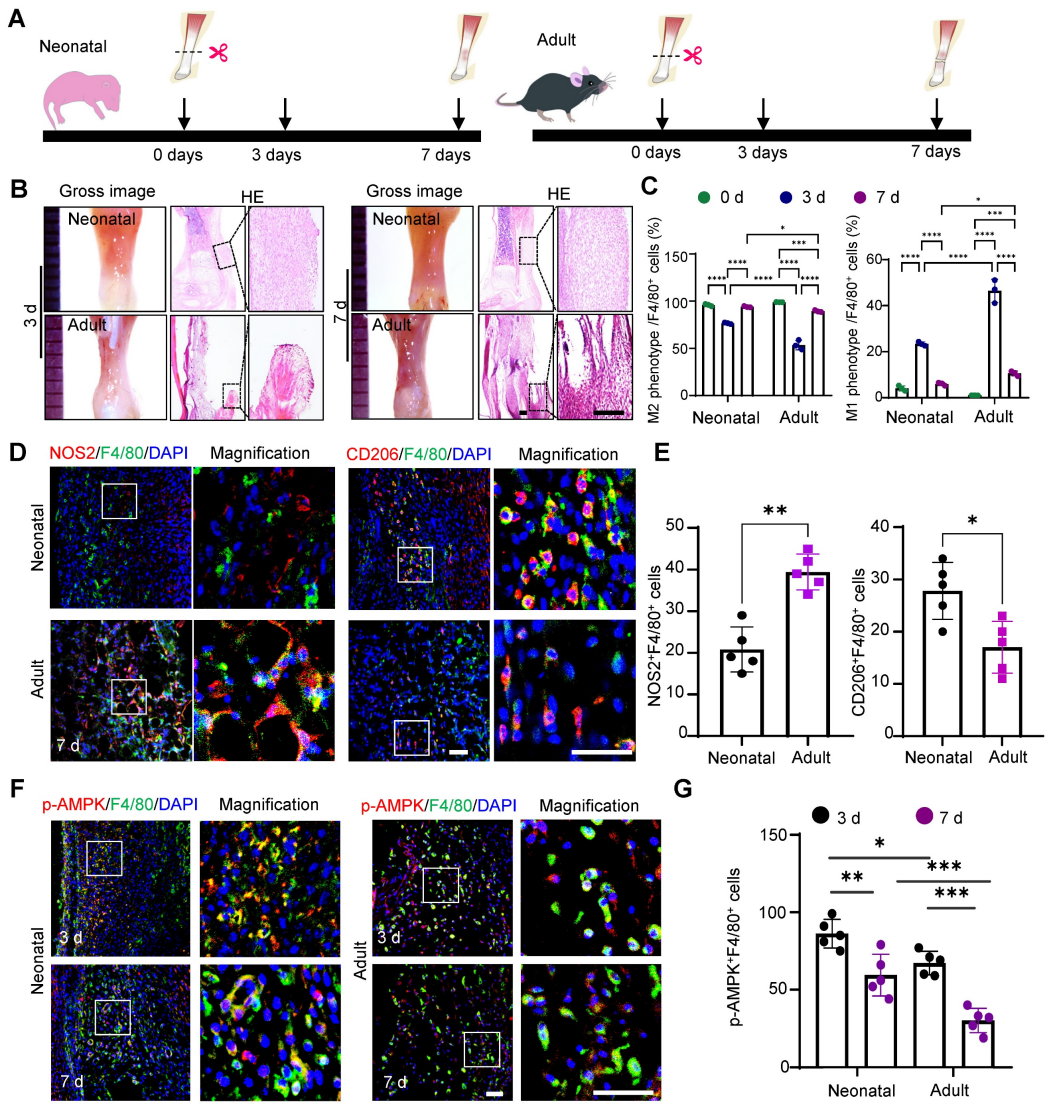
Differential activation of macrophage AMPKα1 during neonatal and adult tendon injury processes. **(A)** Schematic image of the transected tendon model in newborn and adult mice. **(B)** Gross view and HE staining of tendon in newborn and adult mice at 3 and 7 days after injury. Scale bars: 200 µm. **(C)** Quantitative flow cytometry results of M1 (CD45^+^CD11b^+^F4/80^+^CD206^-^) and M2 (CD45^+^CD11b^+^F4/80^+^CD206^+^) in the tendon tissue of newborn and adult mice at 0, 3 and 7 days after injury (*n* = 3). **(D)** Immunofluorescent co-staining of NOS2 or CD206 (red) with F4/80 (green) in tendon derived from newborn and adult mice at 7 days after injury. Scale bars: 50 µm. **(E)** Left: number of NOS2 and F4/80 double-positive cells among F4/80-positive cells. Right: number of CD206 and F4/80 double-positive cells among F4/80-positive cells at 7 days (*n* = 5). **(F)** Immunofluorescent co-staining of p-AMPK (red) with F4/80 (green) in tendon derived from newborn and adult mice at 3 and 7 days after injury. Scale bars: 50 µm. **(G)** Number of p-AMPK and F4/80 double-positive cells among F4/80-positive cells at 3 and 7 days after injury (*n* = 5). * p < 0.05, **p < 0.01, ***p < 0.001 and ****p < 0.0001.

To investigate the differential macrophage responses to tendon injury between the neonatal and adult stages, we established a tendon transection model in neonatal (0-1 days) and adult (12 weeks) mice (Fig. 1A) 28. Seven days after injury, the neonatal tendon tissue exhibited superior healing compared to the adult group, characterized by a relatively orderly collagen fiber arrangement and a significant decrease in inflammatory cells compared to that on day 3. In contrast, adult tendon tissue displayed marked collagen fiber disruption, prominent adhesion at the ends, and evident inflammatory infiltration (Fig. 1B). The switch from the pro-inflammatory M1 phenotype to the anti-inflammatory M2 phenotype is essential for the transition from an initial injury response to the tissue repair phase. Flow cytometric analysis indicated that neonatal mice exhibited earlier activation of the anti-inflammatory cluster of M2 macrophages (CD45^+^CD11b^+^F4/80^+^CD206^+^) and more rapid recession of pro-inflammatory M1 (CD45^+^CD11b^+^F4/80^+^CD206^-^) macrophages (Fig. 1C and Fig. S1). Immunofluorescence staining also confirmed that 7 days after injury, the percentage of anti-inflammatory macrophages (~29% CD206^+^F4/80^+^) in the tendon tissues of neonatal mice was significantly higher than that in adult mice (~18% CD206^+^F4/80^+^). Conversely, the neonatal group had significantly fewer pro-inflammatory macrophages (~19% NOS2^+^F4/80^+^) than the adult group (~39% NOS2^+^F4/80^+^) (Fig. 1D, E).

AMPKα1 serves as a critical regulator of macrophage metabolism, function, and polarization 29. Therefore, we examined the AMPKα1 expression in macrophages at 3- and 7-days post-injury. The results revealed significant AMPK activation in macrophages, with a higher number of phosphorylated AMP-activated protein kinase (p-AMPK)-positive cells in neonatal mice than in adult mice (Fig. 1F, G). Taken together, the* in vivo* findings highlight the variations in healing outcomes and macrophage phenotypes between neonatal and adult mice, implying that the differential activation of AMPK in macrophages during tendon injury may play a role in regulating macrophage phenotypes and orchestrating tendon regeneration.

### AMPKα1 deficiency impedes the transition of macrophage to an anti-inflammatory phenotype

Next, to determine whether AMPKα1 deficiency affects macrophage functionality, we generated the myeloid-specific *Ampkα1* knockout mice (*LysM-Cre*; *Ampkα1^fl/fl^*) and used their littermate *Ampk^fl/fl^* mice as controls. First, the genotyping PCR and western blotting results verified the knockout efficiency at both the genomic and protein levels (Fig. S2A, B). The relationship between AMPKα1 activation and macrophage function was also investigated *in vitro*. Primary BMDMs obtained from *Ampk^fl/fl^* and *LysM-Cre; Ampkα1^fl/fl^* mice. Furthermore, lipopolysaccharide (LPS) and interleukin 4 (IL-4) were used to induce the polarization of BMDMs toward M1 pro-inflammatory and M2 inflammatory-resolving phenotypes, respectively. BMDMs from *LysM-Cre; Ampkα1^fl/fl^* mice exhibited increased expression of the M1 macrophage markers NOS2 and tumor necrosis factor alpha (TNF-α), and decreased expression of the M2 macrophage markers YM1 and CD206 at both the gene and protein levels (Fig. S3A-D). Furthermore, we observed significant differences in cytokine secretion by macrophages between *Ampkα1^fl/fl^* and *LysM-Cre; Ampkα1^fl/fl^* mice. Specifically, the levels of anti-inflammatory cytokines IL-4 and IL-10 were significantly reduced, whereas the levels of pro-inflammatory cytokines IL-6 and TNF-α were markedly elevated (Fig. S3E). Efferocytosis is another crucial function of macrophages in maintaining homeostasis by clearing apoptotic cells 30. Here, we observed that the capacity of phagocytosis was significantly reduced in AMPKα1-deficient macrophages. Specifically, BMDMs from *LysM-Cre; Ampkα1^fl/fl^* mice showed a lower phagocytic index (~42%) than BMDMs from* Ampk^fl/fl^* (~19%) mice (Fig. S3F). These results suggested that the absence of AMPKα1 in macrophages impedes their transition to an anti-inflammatory phenotype and significantly diminishes their phagocytic activity.

### Macrophage AMPKα1 deficiency impairs tendon repair and regeneration

**Figure 2:**
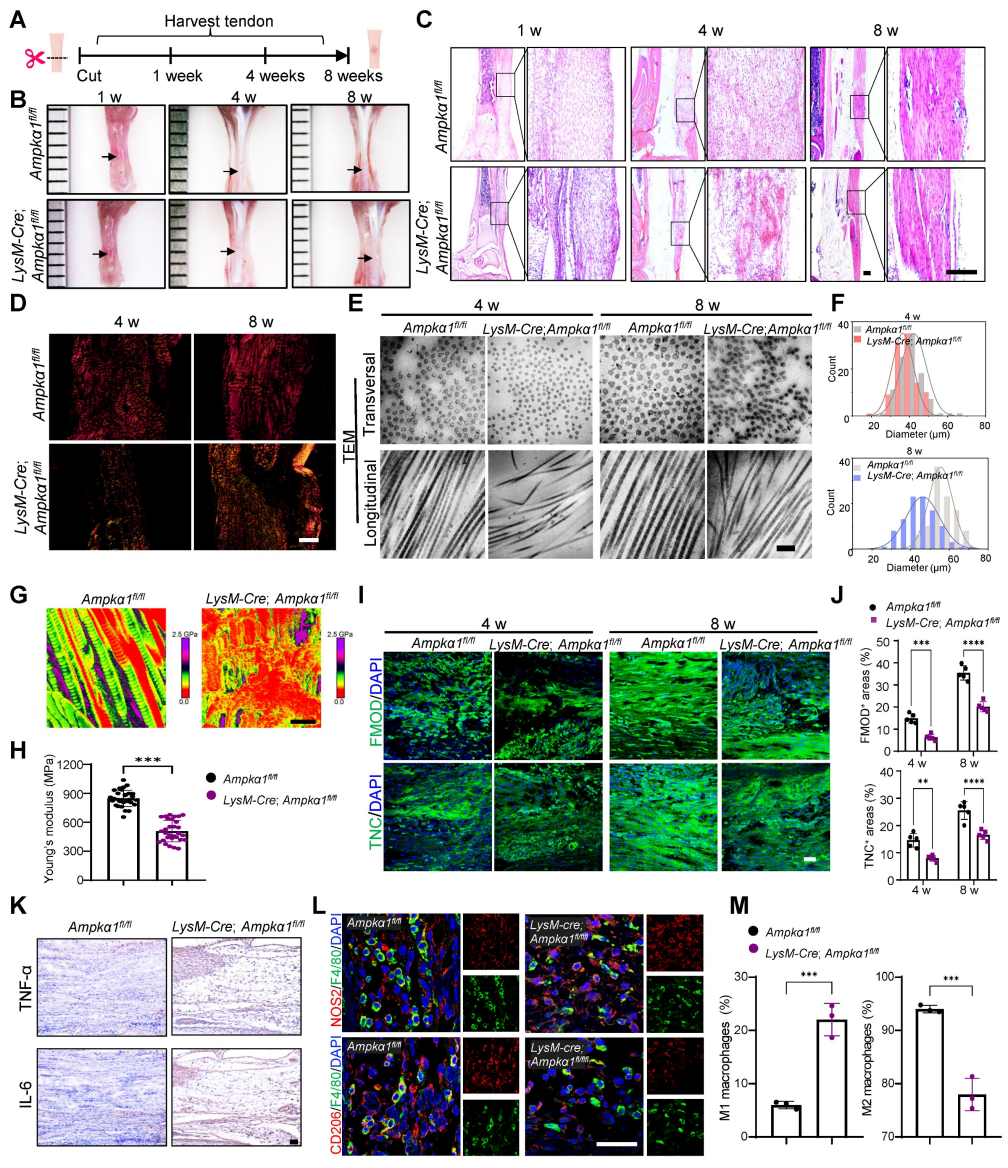
Macrophage AMPKα1 deficiency impairs tendon regeneration and repair capacity.** (A)** Scheme depicting of construction of the *LysM-Cre; Ampkα1^fl/fl^* mice and littermate control mice (*Ampkα1^fl/fl^*), along with a schematic image of the transected tendon model. **(B)** Gross view of tendon from *LysM-Cre; Ampkα1^fl/fl^* and control mice at 1-, 4- and 8-week after injury. Black arrows point to areas of tendon tissue damage.** (C)** Representative HE staining of the tendon from *LysM-Cre; Ampkα1^fl/fl^* and control mice at 1-, 4- and 8-week postoperatively. Scale bars: 200 µm. **(D)** Representative Sirius red staining images of tendon from *LysM-Cre; Ampkα1^fl/fl^* and control mice at 4 and 8 weeks postoperatively, imaged using a polarized light microscope. Type I fibers (thick fibers, yellow-orange); Type III fibers (thin fibers, green). Scale bars: 100 µm. **(E)** Representative transmission electron microscopic (TEM) images illustrating the microstructural characteristic of regenerated collagen fibers in the control and* LysM-Cre; Ampkα1^fl/fl^* group at 4 and 8 weeks after injury. Scale bars: 200 nm. **(F)** Frequency distribution profiles of fibril diameter in the tendon of control and* LysM-Cre; Ampkα1^fl/fl^* group at 4 and 8 weeks after injury. **(G)** Representative atomic force microscope images of the tendon from *LysM-Cre; Ampkα1^fl/fl^* and control mice at 8-week postoperatively. Scale bars: 400 nm.** (H)** Young's modulus of the tendon from *LysM-Cre; Ampkα1^fl/fl^* and control mice. **(I)** Immunofluorescent staining of TNC and FMOD in tendon derived from control and *LysM-Cre; Ampkα1^fl/fl^* mice at 4 and 8 weeks after injury. Scale bars: 50 µm. **(J)** Quantitative analysis of immunofluorescence staining of FMOD and TNC (*n* = 5).** (K)** Immunohistochemical staining of IL-6 and TNF-α in tendon derived from control and *LysM-Cre; Ampkα1^fl/fl^* mice at 1 week after injury. Scale bars: 200 µm. **(L)** Immunofluorescent detection of F4/80 and CD206 in tendon derived from control and *LysM-Cre; Ampkα1^fl/fl^* mice at 1 week after injury. Scale bars: 50 µm. **(M)** Flow cytometry analysis of the percentage of CD45^+^CD11b^+^F4/80^+^CD206^+^ macrophages and CD45^+^CD11b^+^F4/80^+^CD206^-^ macrophages in the tendon of control and *LysM-Cre; Ampkα1^fl/fl^* mice at 1 week after injury (*n* = 3). **p < 0.01, ***p < 0.001, and ****p < 0.0001.

To further investigate the influence of macrophage AMPKα1 loss on neonatal tendon regeneration, tendon transection models in *LysM-Cre*; *Ampkα1^fl/fl^* neonatal mice were established, and samples were harvested at 1-, 4-, and 8-weeks post-injury, respectively (Fig. 2A). The results revealed that, compared to the control group, the tendon repair area in the *LysM-Cre; Ampkα1^fl/fl^* group exhibited poorer smoothness and flatness, with translucent collagen tissue deposition and reduction in overall tendon length at 4- and 8-weeks post-operation (Fig. 2B and Fig. S4A). At 1-week post-injury, disorganized and scattered fiber were observed in the *LysM-Cre; Ampkα1^fl/fl^* group. Similarly, compared to the control group, the regenerated tendon collagen fibers in the *LysM-Cre; Ampkα1^fl/fl^* mice appeared looser, more disorganized, and less mature at 4 and 8 weeks post-injury (Fig. 2C, D). To further compare the collagen fibril distribution and diameter at the nanoscale level, specimens were observed under TEM and SEM (Fig. 2E, F and Fig. S4B). Representative images revealed increased collagen fibril entanglement and disorganization in *LysM-Cre; Ampkα1^fl/fl^* group at 4- and 8-weeks post-operation. To assess the relationship between fibril pattern and tendon mechanics, AFM was performed to examine the fibrillar crimp and Young's modulus of the regenerated tendons (Fig. 2G, H). The average Young's modulus was ~508 MPa in the *LysM-Cre; Ampkα1^fl/fl^* group, compared to ~847 MPa in the control group. These findings indicate that *Ampkα1* expression in macrophages is both necessary and sufficient for the regeneration of tendons with competent tensile mechanical properties.

During tendon injury, multiple tendon-resident cells coordinate with macrophages in the inflamed tissue to achieve efficient repair 31. Scleraxis-lineage (Scx^Lin^) cells are vital during the process of neonatal tendon repair 32 and may include tendon progenitor cells and differentiated tenocytes. Compared to the control group, a significant decrease in the percentage of Ki67^+^SCX^+^ cells and AMPK^+^F4/80^+^ macrophages were observed in the *LysM-Cre; Ampkα1^fl/fl^* group at 1 and 4 weeks post-injury (Fig. S4C, D). This preliminary finding suggested an impaired proliferation capacity of tendon cells in *LysM-Cre; Ampkα1^fl/fl^* mice, potentially resulting from macrophage AMPKα1 deficiency. Additionally, histological analysis at 4- and 8-weeks post-injury revealed markedly reduced expression of tendon-related factors fibromodulin (FMOD) and tenascin-C (TNC), along with more disordered nuclear arrangement in the *LysM-Cre; Ampkα1^fl/fl^* group compared to the control group (Fig. 2I, J), suggesting impaired regenerative and differentiation capacity of tendon cells. Given that AMPKα1 phosphorylation may favor anti-inflammatory macrophage polarization, we further examined the inflammatory profile by analyzing canonical M1/M2 macrophage markers and pro-inflammatory cytokines IL-6 and TNF-αand anti-inflammatory cytokines TGF-β and IL-10 (Fig. 2K-M and Fig. S4E, F). Compared with the control group, the number of pro-inflammatory M1 macrophages cells and the level of pro-inflammatory factors IL-6 and TNFα increased significantly in the *LysM-Cre; Ampkα1^fl/fl^* mice, while the expression of anti-inflammatory cytokines (TGF-β and IL-10) was significantly reduced. Together, these findings imply that loss of *Ampkα1* in macrophages disrupts the inflammatory microenvironment, impairs tendon cell function, and consequently compromises tendon regenerative capacity.

### Ampkα1 deletion in macrophages impairs TSPC function

TSPCs are a vital population of tendon-resident cells. During tendon regeneration and repair, macrophages interact with TSPCs to modulate their proliferation, differentiation, and migration, which are crucial for effective tendon regeneration 33. To understand how AMPKα1 deficiency in macrophages delays tendon repair, we first explored the potential functional alteration of TSPCs from *LysM-Cre; Ampkα1^fl/fl^* mice. We collected conditioned media from BMDMs of *Ampkα1^fl/fl^* and *LysM-Cre; Ampkα1^fl/fl^* mice and used them to treat TSPCs (Fig. S5A). Immunofluorescence staining results showed that the TPSCs that were stimulated by the conditioned medium from *LysM-Cre; Ampkα1^fl/fl^* BMDMs displayed lower proliferation capacity (Fig. S5B, C). Correspondingly, there was a notable decrease in the colony-forming capacity of TSPCs *in vitro* following stimulation with conditioned medium from *LysM-Cre; Ampkα1^fl/fl^* BMDMs (Fig. S5D, E). Subsequently, we assessed the effects of conditioned media from both macrophage groups on TSPC migration using a scratch assay. The results demonstrated that the migration rate of TSPCs was reduced when treated with conditioned medium from the *LysM-Cre; Ampkα1^fl/fl^* group compared to that from the control group (Fig. S5F, G).

Furthermore, we induced tenogenic differentiation of TSPCs and supplemented the differentiation medium with conditioned medium from macrophages. The RT-qPCR results showed that TSPCs stimulated by the conditioned medium from *LysM-Cre; Ampkα1^fl/fl^* BMDMs displayed a significant decrease in the mRNA expression of the tendon-related genes *collagen I* (*Col1*), *Tnmd*, and *Tnc* (Fig. S5H). Additionally, the Sirius Red staining results demonstrated that TSPCs stimulated with conditioned medium from *LysM-Cre; Ampkα1^fl/fl^* BMDMs, also exhibited lower collagen matrix secretion (Fig. S5I, J). These *in vitro* findings further confirmed that the activation of AMPKα1 in macrophages can disturb the harmonious communication between the TSPCs and macrophages, which impairs multiple biological functions of TPSCs and contributes to the decreased tendon regenerative capacity in *LysM-Cre; Ampkα1^fl/fl^* mice.

### Ampkα1-deficient macrophages promote FBP2 upregulation in TSPCs

**Figure 3:**
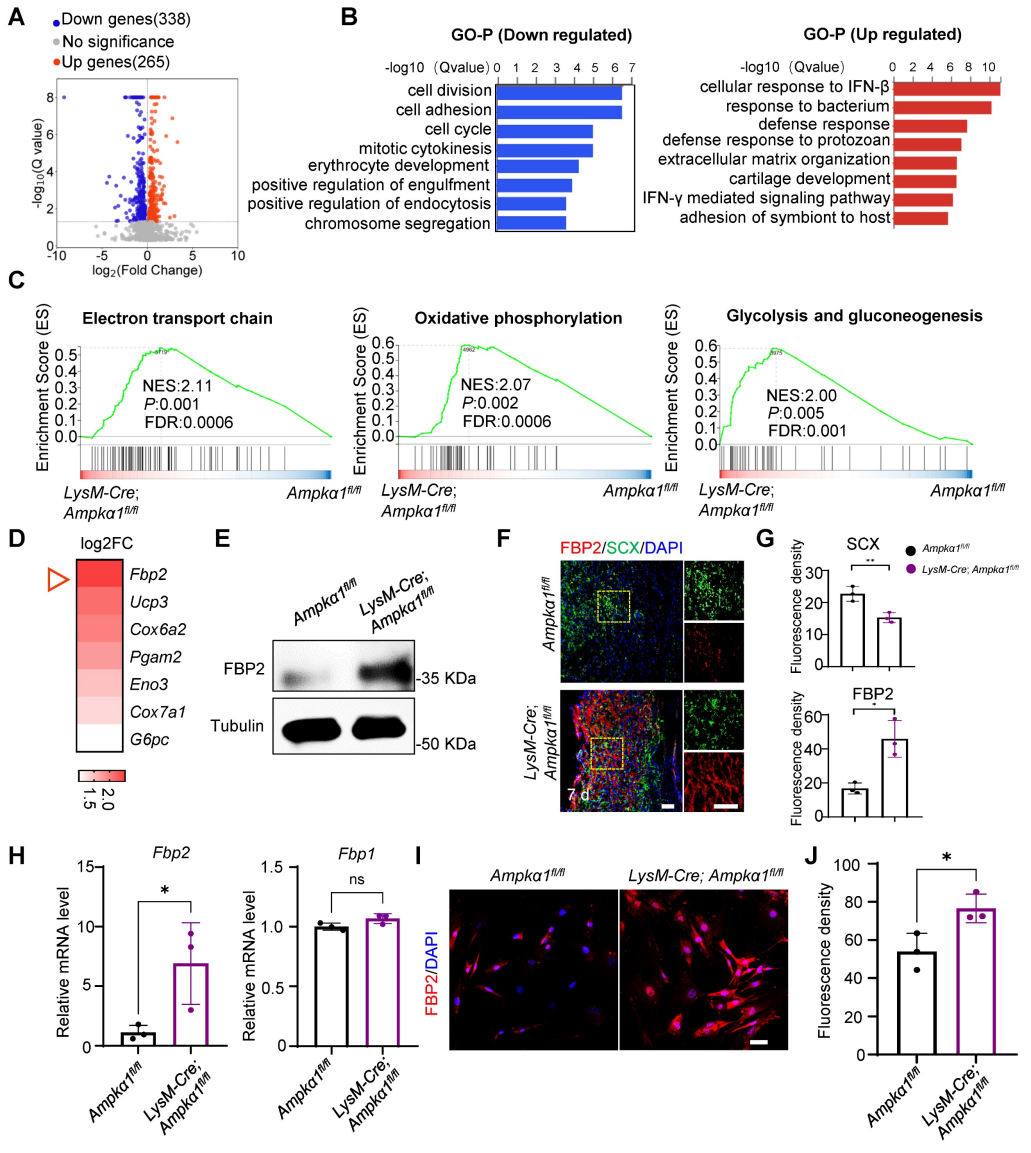
The enhanced expression of FBP2 in tendon tissue of *LysM-Cre; Ampkα1^fl/fl^* mice after injury. **(A)** Volcano plots visualizing the gene expression for up/down-regulated genes in *LysM-Cre; Ampkα1^fl/fl^* tendon tissue relative to control tissue after injury (*n* = 3). **(B)** Gene Ontology Biological Process (GO-BP) analysis of down- (left) and upregulated (right) genes (Top 10 biological pathways). **(C)** GSEA enrichment curves (based on WikiPathways database) depicting the three most significant enriched upregulated pathways. **(D)** The heatmap displaying genes ranked by log_2_FC values in (C). **(E)** Western blotting analysis of FBP2 expression in the tendon tissue of control and *LysM-Cre; Ampkα1^fl/fl^* mice after injury. **(F)** Location of FBP2 in the TSPCs by immunofluorescence staining.** (G)** Quantification of FBP2 and SCX fluorescent intensity (*n* = 3). **(H)**The expression of *Fbp2*, and *Fbp1* in TSPCs was evaluated by RT-PCR analysis (*n* = 3). **(I)** Immunofluorescence staining results showed the protein expression levels of FBP2 (red) in TSPCs derived from *LysM-Cre; Ampkα1^fl/fl^* group, compared to the control TSPCs. Scale bars: 20 µm. **(J)** Quantification of FBP2 fluorescent intensity (*n* = 3). ns: no significance; *p < 0.05 and **p < 0.01.

To gain further insights into the underlying mechanism of macrophage AMPKα1 in tendon regeneration, we performed RNA sequencing on tissues from *LysM-Cre; Ampkα1^fl/fl^* mice and *Ampkα1^fl/fl^* mice 7 days after injury. Initial transcriptome analysis revealed a total of 603 differentially expressed genes between the *LysM-Cre; Ampkα1^fl/fl^* group and *Ampkα1^fl/fl^* group. Among these, 338 genes were downregulated, and 265 genes were upregulated (Fig. 3A). Subsequent Gene Ontology (GO) analysis of the upregulated and downregulated genes revealed significant enrichment of differentially expressed genes in categories such as cell division, cell proliferation, and cellular immune response (Fig. 3B). Additionally, Gene Set Enrichment Analysis (GSEA) pinpointed the “electron transport chain,” “oxidative phosphorylation,” and “glycolysis and gluconeogenesis” as key pathways involved in the upregulation of genes in the *LysM-Cre; Ampkα1^fl/fl^* group post-injury (Fig. 3C). We further investigated the genes with significant differential expression (log_2_FC >1) in these three pathways. Notably, *Fbp2* ranked first in the differential expression of upregulated genes in the *LysM-Cre; Ampkα1^fl/fl^* group (Fig. 3D).

A previous study indicated that FBP2 is a key enzyme that limits gluconeogenesis, antagonizes glycolysis, and reduces glucose uptake 23. Validation through western blotting and immunofluorescence staining confirmed that a consistent upregulation of FBP2 protein expression within tendon tissues of the *LysM-Cre; Ampkα1^fl/^*^fl^ group (Fig. 3E, F). Meanwhile, we observed the co-localization of FBP2 and tendon-specific markers (SCX, CD146, and CD90) in damaged tissues, which are markers of TSPCs (Fig. 3F, G and Fig. S6). The results revealed that, compared to the *Ampkα1^fl/fl^* group, FBP2 expression was significantly upregulated in the *LysM-Cre; Ampkα1^fl/fl^* group after tendon injury. To further investigate FBP2 expression specifically in TSPCs, we isolated cells from injured tendon tissues in both groups. We confirmed that *Fbp2* was upregulated in the TSPCs of the *LysM-Cre; Ampkα1^fl/fl^* group at the mRNA level compared to controls, while no obvious change at the gene level was detected in *Fbp1*, an isozyme of FBP2 (Fig. 3H). Consistently, immunofluorescence staining certified markedly higher FBP2 protein levels in TSPCs from the *LysM-Cre; Ampkα1^fl/fl^* group than in controls (Fig. 3I, J).

### Elevated TNF-α in tendon niche increased FBP2 levels in a PI3K/AKT-dependent manner

**Figure 4:**
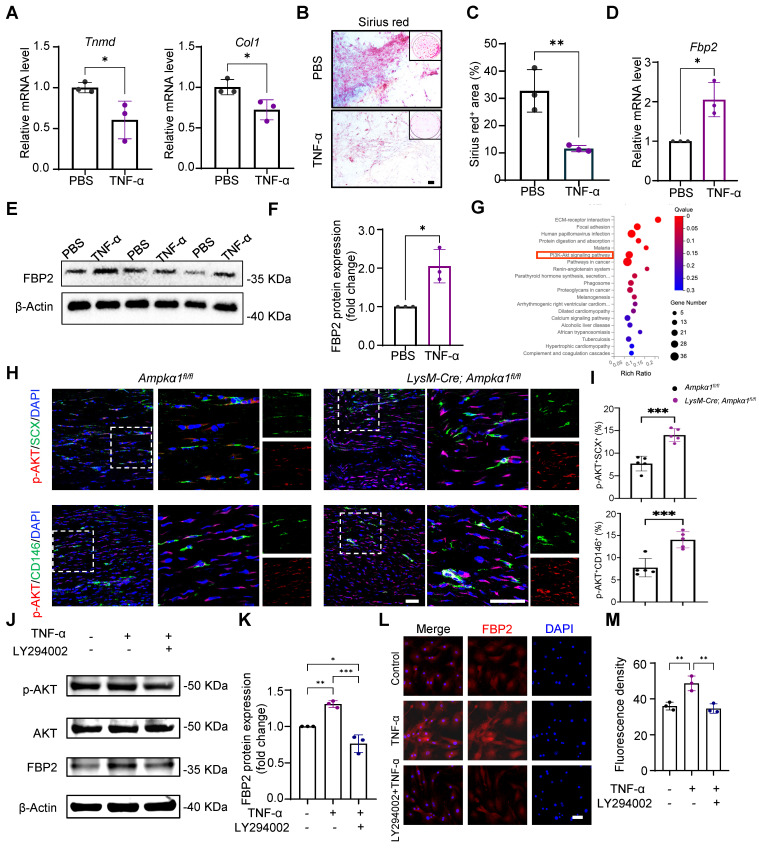
TNF-α increased FBP2 levels through PI3K /AKT signaling.** (A)** The expression of *Tnmd*, and *Col1* in TSPCs stimulated by TNF-α was evaluated by RT-PCR analysis (*n* = 3). **(B)** Photomicrographs of Sirius red staining of TSPCs stimulated by TNF-α for 10 days after tenogenic differentiation induction. Scale bars: 200 µm. **(C)** Quantification of Sirius red-positive areas (*n* = 3). **(D)** The expression of *Fbp2* in TSPCs stimulated by TNF-α was evaluated by RT-PCR analysis (*n* = 3). **(E)** Western blotting analysis of FBP2 expression in TSPCs stimulated by TNF-α. **(F)** Quantification of western blotting results in (E). **(G)** Kyoto Encyclopedia of Genes and Genomes (KEGG) analysis of upregulated genes of *LysM-Cre; Ampkα1^fl/fl^* group compared with control group. **(H)** Immunofluorescence staining results showed the protein expression levels of p-AKT (red) in TSPCs (green) of *LysM-Cre; Ampkα1^fl/fl^* group and control group. Scale bars: 50 µm. **(I)** Quantification analysis of p-AKT^+^SCX^+^-positive cells and p-AKT^+^CD146^+^-positive cells (*n* = 5). **(J)** Western blotting analysis of p-AKT, AKT and FBP2 expression in TSPCs stimulated by TNF-α and/or LY294002. **(K)** Quantification of western blotting results in (J) (*n* = 3). **(L)** Immunofluorescence staining results showed the protein expression levels of FBP2 (red) in TSPCs stimulated by TNF-α and/or LY294002. Scale bars:50 µm. **(M)** Quantification of FBP2 fluorescent intensity (*n* = 3). *p < 0.05, **p < 0.01 and ***p < 0.001.

Macrophages primarily modulate tissue repair through paracrine cytokines. Consistent with this, the above results showed that tendon and BMDMs from *LysM-Cre; Ampkα1^fl/fl^* mice displayed the persistently elevated levels of the pro-inflammatory factors TNF-α and IL-6 (Fig. 2K and Fig. S4E, F). Notably, our previous study demonstrated that TNF-α stimulation impairs the differentiation capacity of TSPCs 28. Build on this observation, we next investigated whether TNF-α drives the TSPC dysfunction seen in AMPKα1-deficient mice and simultaneously probed the downstream signaling mechanisms underlying this effect. Upon TNF-α treatment, mRNA expression of tendon marker genes *Tnmd* and *Col1* significantly decreased (Fig. 4A). Sirius Red staining also showed that TNF-a stimulation reduced the collagen matrix secretion (Fig. 4B, C).

Simultaneously, we found that exposure to TNF-α markedly increased FBP2 expression in TSPCs relative to untreated controls (Fig. 4D-F). Kyoto Encyclopedia of Genes and Genomes (KEGG) analysis further showed that genes upregulated in the *LysM-Cre; Ampkα1^fl/fl^* tendons were significantly enriched in the PI3K/AKT signaling pathway (Fig. 4G). Meanwhile, immunofluorescent staining results confirmed that compared to the *Ampkα1^fl/fl^* group, AKT activation of TSPCs was significantly increased in the *LysM-Cre; Ampkα1^fl/fl^* group after tendon injury (Fig. 4H, I). It has been shown that TNF-α can drive metabolic reprogramming by promoting signaling through PI3K/AKT, which deteriorates the stem-cell state and cellular differentiation 34, 35. To explore whether TNF-α affects FBP2 expression through the PI3K/AKT pathway, we applied the PI3K/AKT pathway inhibitor LY294002. The results revealed that PI3K/AKT was significantly inhibited by LY294002, and the expression of FBP2 was simultaneously inhibited (Fig. 4J-M). To directly validate the essential role of TNF-α in the proposed signaling axis, we have now performed additional rescue experiments using a TNF-α neutralizing antibody. Specifically, conditioned medium from AMPKα1-deficient macrophages was treated with an anti-TNF-α antibody prior to incubation with TSPCs. The results demonstrate that neutralization of TNF-α markedly attenuates the upregulation of FBP2 induced by AMPKα1-deficient macrophage-conditioned medium (Fig. S7A-D). Taken together, these findings indicate that AMPKα1 deletion in macrophages during tendon repair leads to high TNF-α expression, whereas the resulting high TNF-α levels within the tendon niche further cause AKT activation and FBP2 overexpression, deteriorating the tendon stem cell state.

### Knockdown of Fbp2 enhances TSPC function *in vitro*

**Figure 5:**
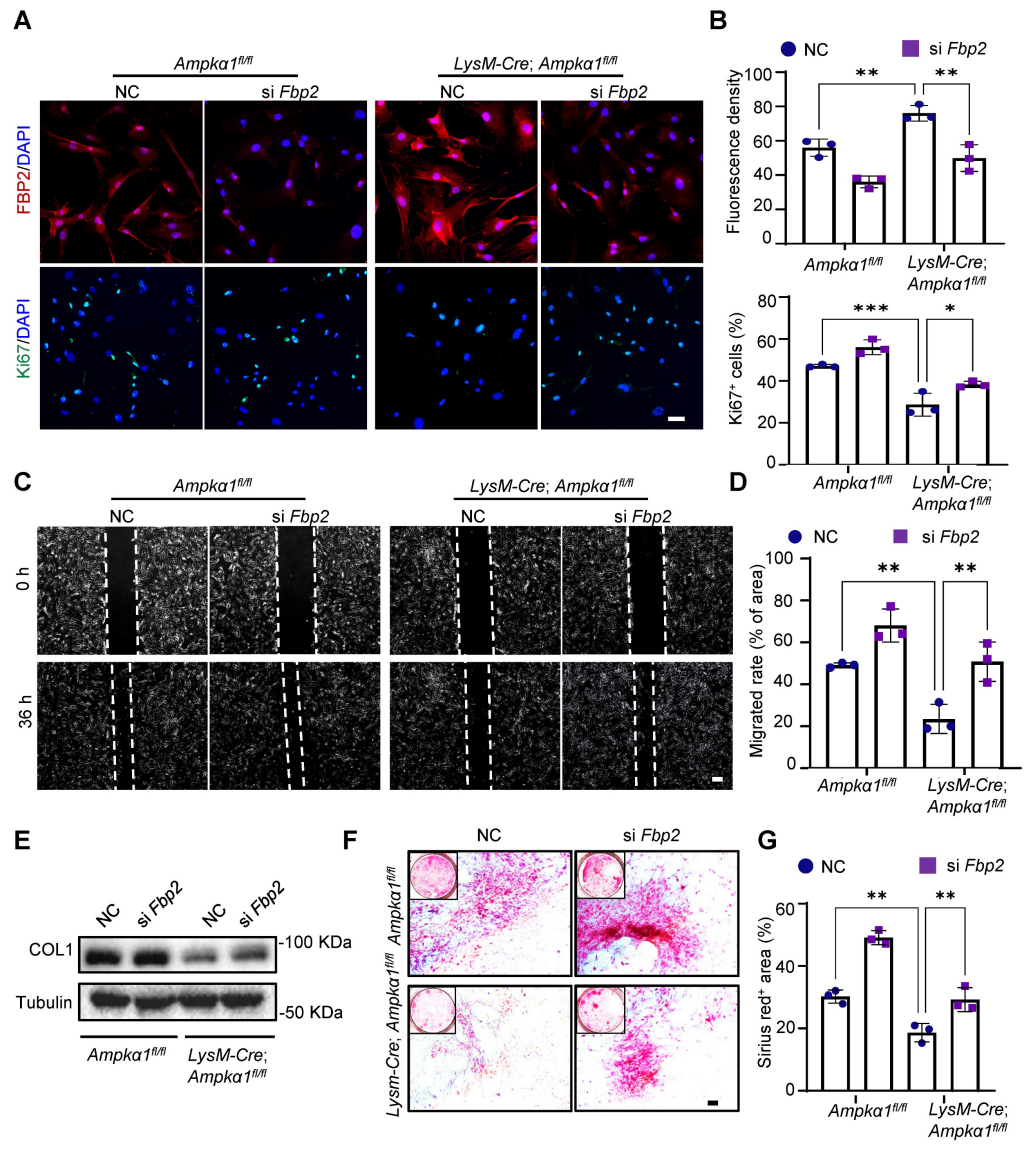
Knockdown of *Fbp2* promotes proliferation, migration, and differentiation capacity of TSPCs *in vitro*. **(A)** Immunofluorescence staining results showed the protein expression levels of FBP2 (red) and Ki67 (green) in Fbp2-knockdown TSPCs, compared to NC group. Scale bars: 20 µm. **(B)** Quantification of ratio of Ki67 positive cells and FBP2 fluorescent intensity (*n* = 3). **(C)** Representative images of the wound-healing assay of the TSPCs transfected with si *Fbp2* at 0 h and 36 h post-scratch. Scale bars: 200 µm. **(D)** The percentage of recovered area in scratch assay (*n* = 3). **(E)** Western blotting analysis of COL1 expression in TSPCs transfected with si *Fbp2*. **(F)** Photomicrographs of Sirius red staining of TSPCs transfected with si *Fbp2* were cultured for 10 days after tenogenic differentiation induction, the collagen fibers were stained red. Scale bars: 200 µm. **(G)** Quantification of Sirius red-positive areas (*n* = 3). *p < 0.05, **p < 0.01, and ***p < 0.001.

We explored the regulatory role of FBP2 in proliferation, migration, and differentiation. We utilized small interfering RNA (siRNA) to silence the expression of *Fbp2* in TSPCs (si *Fbp2*). siRNA (NC) was used as a control. Preliminary experiments confirmed that siRNA treatment significantly reduced FBP2 levels (Fig. S8A, B). Then, immunofluorescence staining results demonstrated that *Fbp2* knockdown significantly increased the proportion of Ki67^+^ cell proportions in both *LysM-Cre; Ampkα1^fl/fl^* TSPCs and *Ampkα1^fl/fl^* group, thereby enhancing cell proliferation capacity (Fig. 5A, B). Moreover, wound healing experiments indicated that the knockdown of *Fbp2* accelerated TSPC growth after scratching (Fig. 5C, D). Subsequently, the tenogenic differentiation capacity of TSPCs after knockdown was assessed. Western blotting results revealed that there was a remarkable declination in COL1 protein expression in TSPCs from* LysM-Cre; Ampkα1^fl/fl^* mice, which was reversed by the *Fbp2* knockdown (Fig. 5E). Furthermore, Sirius Red staining results demonstrated that the *Fbp2* knockdown remarkably restore the impaired tenogenic differentiation capacity of TSPCs from *LysM-Cre; Ampkα1^fl/fl^* mice, as evidenced by a significant increase in the proportion of positively stained areas in cells transfected with si *Fbp2* (Fig. 5F, G). Collectively, these findings suggest that the knockdown of *Fbp2* enhances the proliferation, migration, and tendon differentiation of TSPCs.

### FBP2 leads to mitochondrial dysfunction and suppression of mitochondrial biogenesis

To address whether the effects of FBP2 on TSPCs are mediated by its enzymatic activity and downstream metabolites, we first focused on its canonical catalytic function. Specifically, we measured the levels of fructose-6-phosphate (F-6-P), the direct metabolic product of FBP2, and confirmed that FBP2 overexpression significantly increased intracellular F-6-P levels (Fig. S9A). To further determine whether this metabolite mediates the biological effects of FBP2, we examined the effects of exogenous F-6-P on TSPC proliferation and differentiation. However, exogenous supplementation of F-6-P to TSPCs did not significantly affect their proliferation or differentiation capacity, indicating that FBP2 may exert its effects through a non-enzymatic mechanism (Fig. S9 B-D). Indeed, emerging evidence suggests that FBP2 can regulate mitochondrial function via protein-protein interactions independent of its catalytic activity 36. To elucidate the specific mechanism by which FBP2 regulates TSPC function, we compared the mitochondrial biogenesis and function of TSPCs in *LysM-Cre; Ampkα1^fl/fl^* and *Ampkα1^fl/fl^* group and investigated the impact of *Fbp2* knockdown on TSPC mitochondria. Mitochondrial visualization of TSPCs and mitochondrial DNA content assays were performed.

**Figure 6:**
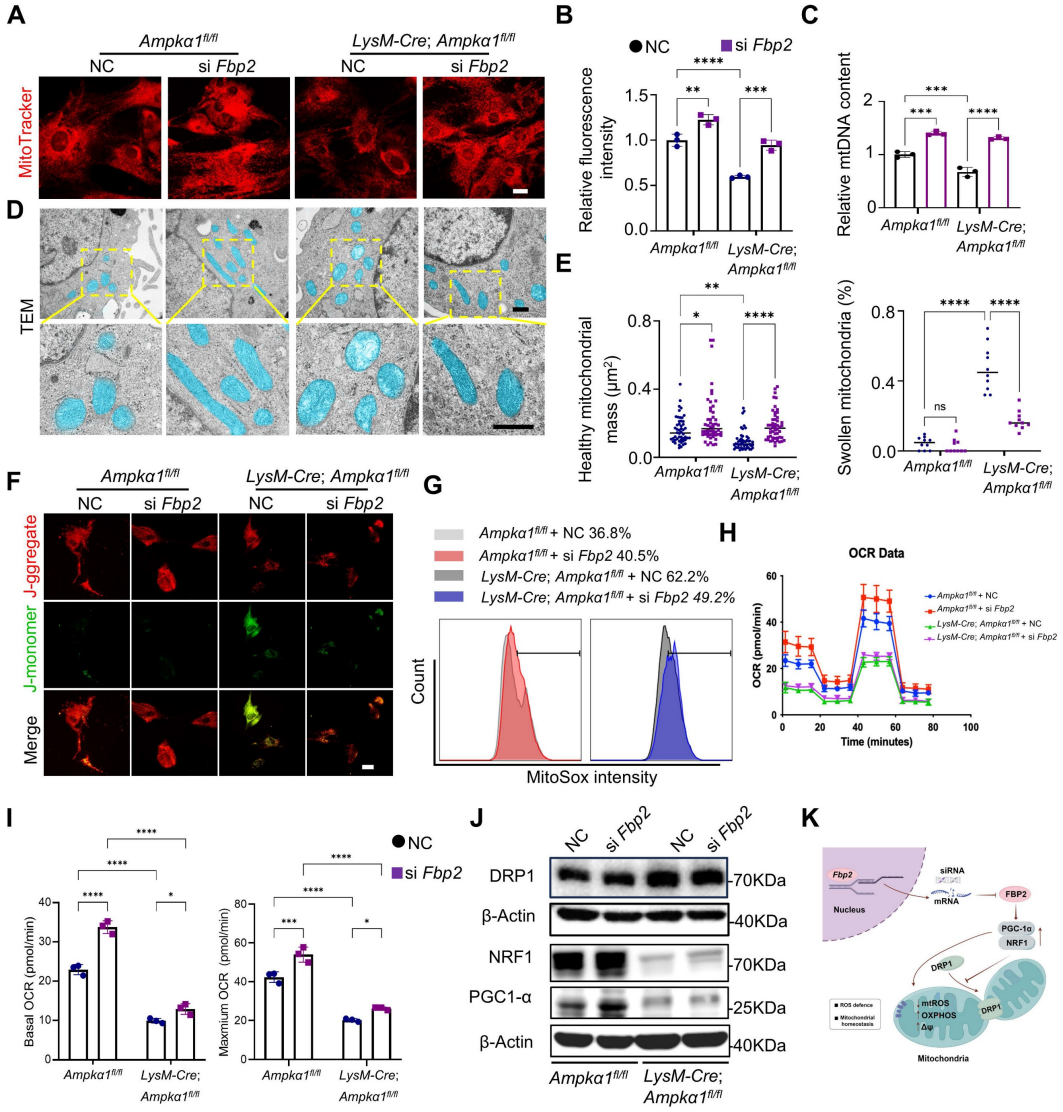
Downregulation of FBP2 promotes mitochondrial biogenesis and functions.** (A)** TSPCs transfected with si NC and si *Fbp2* were stained with MitoTraker Red. Scale bars: 50 µm. **(B)** Quantitative analysis of fluorescence staining of MitoTracker (*n* = 3). **(C)** mtDNA content was determined by quantitative PCR (*n* = 3). mtDNA: mitochondrial DNA. **(D)** Pseudo-colored serial TEM images of TSPCs transfected with si NC and si *Fbp2.* Blue: mitochondria. Below: magnified view of yellow frame. Scale bars: 500 nm. **(E)** Quantitative analysis of mitochondria. Left: the number of healthy mitochondria. Right: the percentage of swollen mitochondria. **(F)** Representative JC1 fluorescence images of TSPCs transfected with si NC and si *Fbp2.* Red: JC1 dye in aggregated forms. Green: JC1 dye in monomeric forms. Scale bars: 50 µm. **(G)** TSPCs were labeled with MitoSOX Red, and MitoSOX Red fluorescence was analyzed using flow cytometry. **(H)** The levels of mitochondrial oxygen consumption rate (OCR) in TSPCs with different treatment (*n* = 3). **(I)** Quantitative data of OCR (*n* = 3). **(J)** Western blotting analysis of DRP1, NRF1, and PGC-1α expression in TSPCs transfected with si NC and si *Fbp2*. **(K)** Schematic illustration of the mechanism of action of FBP2 at the mitochondria in TSPCs. This figure was created using Figdraw. *p < 0.05, **p < 0.01, ***p < 0.001, and ****p < 0.0001.

The outcomes revealed that, compared to TSPCs of *Ampkα1^fl/fl^* mice, mitochondrial to nuclear DNA ratios were dramatically decreased in those of *LysM-Cre; Ampkα1^fl/fl^* mice (Fig. 6A-C). Simultaneously, *Fbp2* knockdown in TSPCs of *Ampkα1^fl/fl^* mice facilitated mitochondrial DNA and enhanced mitochondrial biogenesis. Furthermore, TEM images showed that most TSPC mitochondria in the *Ampkα1^fl/fl^* group exhibited integrated structures, whereas those in the *LysM-Cre; Ampkα1^fl/fl^* group, with elevated FBP2 expression, showed signs of swollen and fragmented mitochondria. In contrast, *Fbp2* knockdown ameliorated the mitochondrial morphological phenotype and maintained mitochondrial health and quality (Fig. 6D, E). We surmised that the observed variations in mitochondrial mass and function between the different TSPC groups could be attributed to the inflammation-related high reactive oxygen species (ROS) microenvironment, and these changes were significantly attenuated by *Fbp2* knockdown, indicating its protective role against oxidative stress-induced mitochondrial damage.

It was found that TSPCs from *LysM-Cre; Ampkα1^fl/fl^* mice exhibited the higher level of cellular and mitochondrial ROS compared with TSPCs from wild-type (WT) mice and displayed decreased mitochondrial membrane potential. Conversely, *Fbp2* knockdown restored mitochondrial function, as indicated by normalization of the mitochondrial membrane potential and reduced excessive ROS production (Fig. 6F, G and Fig. S10A-D). To further evaluate mitochondrial function, the basal oxygen consumption rate (OCR) was measured using a Seahorse analyzer. We found that the OCR of TSPCs from *LysM-Cre; Ampkα1^fl/fl^* mice were reduced, and knockdown of *Fbp2* significantly elevated the OCR over the control (Fig. 6H, I). To elucidate the molecular mechanisms by which FBP2 regulates mitochondrial biogenesis and function, we examined the expression of mitochondria-related regulators including peroxisomal proliferator-activated receptor gamma coactivator 1 alpha (PGC-1α), nuclear respiratory factor 1 (NRF1) and dynamin-related protein-1 (DRP1). Our findings identified that DRP1-activated mitochondrial damage and fragmentation were alleviated by knockdown of *Fbp2* (Fig. 6J and Fig. S10E). During this process, the knockdown of *Fbp2* also protects against mitochondrial oxidative by activating the PGC-1α/NRF-1 pathway, thereby restoring mitochondrial homeostasis and promoting mitochondrial biogenesis. Overall, these results indicate that FBP2 is a significant contributor to mitochondrial ROS production and subsequent mitochondrial impairment, which in turn adversely affects stem cell function (Fig. 6K).

### Fbp2 knockdown in TSPCs notably enhances tendon regeneration

**Figure 7:**
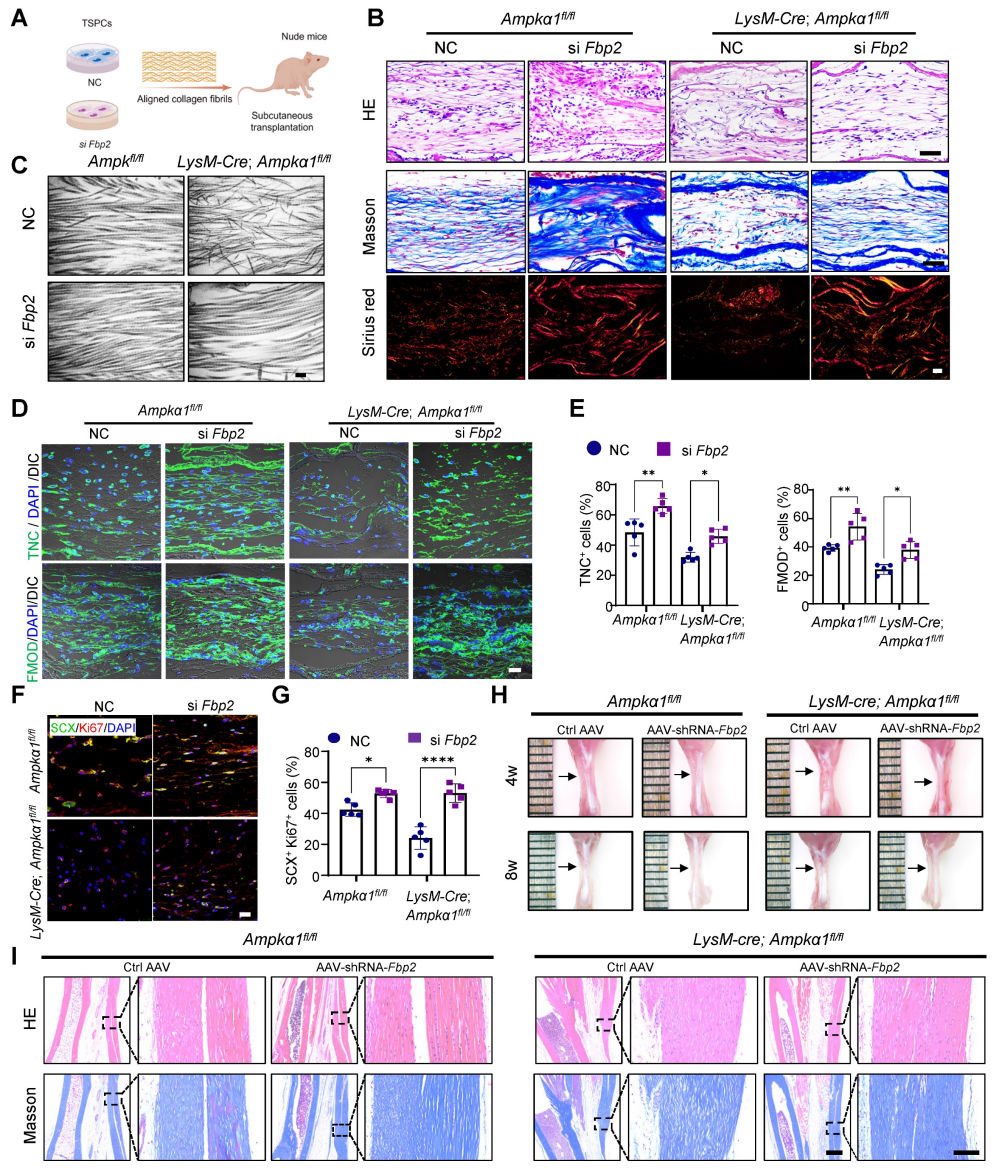
*Fbp2* knockdown in TSPCs notably enhances tendon regeneration. **(A)** Schematic illustration showing TSPCs transfected with si *Fbp2* were loaded onto scaffolds and implanted in the subcutaneous space of nude mice. This figure was created using Figdraw. **(B)** Representative images for HE (top), Masson (middle), and Sirius red staining (polarized light) of newborn tendon-like tissue at 8 weeks after implantation. Scale bars: 50 µm. **(C)** Representative TEM images of newborn tendon-like tissue at 8 weeks after implantation. Scale bars: 200 nm. **(D)** Immunofluorescent staining of TNC and FMOD in newborn tendon-like tissue at 8 weeks after implantation. DIC: differential interference contrast. Scale bars: 20 µm. **(E)** Quantitative analysis of immunofluorescence staining of TNC and FMOD (*n* = 5). **(F)** Immunofluorescent staining of the colocalization of SCX (green) and Ki67 (red) in newborn tendon-like tissue at 8 weeks after implantation. Scale bars: 20 µm. **(G)** Quantitative analysis of immunofluorescence staining of SCX and Ki67 double-positive cells (*n* = 5). **(H)** Gross view of tendon from *LysM-Cre; Ampkα1^fl/fl^* and *Ampkα1^fl/fl^* mice injected with *AAV-Fbp2-shRNA* and control *AAV*-*shRNA* at 4 and 8 weeks after injury. **(I)** Representative HE staining and Masson trichrome staining of the tendon from *LysM-Cre; Ampkα1^fl/fl^* mice and* Ampkα1^fl/fl^* mice injected with *AAV-Fbp2-shRNA* and control *AAV*-*shRNA* at 8 weeks after injury. Low magnification image scale bars: 500 μm; high magnification image scale bars: 100 μm. *p < 0.05, **p < 0.01, and ****p < 0.0001.

To assess the effect of FBP2 on TSPC proliferation and tenogenic capacity *in vivo*, we used a biomimetic parallel collagen scaffold and loaded TSPCs with *Fbp2* knockdown 26*.* These cells were subcutaneously implanted into nude mice for *in vivo* functional validation (Fig. 7A). The grafts were harvested 8 weeks after implantation. HE staining and Masson's trichrome staining revealed that, in both the *Ampkα1^fl/fl^* group and *LysM-Cre; Ampkα1^fl/fl^* group TSPCs with *Fbp2* knockdown*,* the regenerated tendon tissue exhibited notably thicker and denser collagen fibers and deposited more collagen matrix than those in the NC group (Fig. 7B). Furthermore, TEM examination and Sirius Red staining confirmed that knocking down cellular *Fbp2* facilitated the formation of orderly arranged collagen fibers and deposited collagen type I with higher maturity (Fig. 7B, C). Moreover, the si *Fbp2* TSPCs showed a relatively higher proportion of TNC^+^ cells (~66% in *LysM-Cre; Ampkα1^fl/fl^* group; ~45% in* Ampkα1^fl/fl^* group) than the NC group (~48% in *LysM-Cre; Ampkα1^fl/fl^* group; ~32% in* Ampkα1^fl/fl^* group), the si *Fbp2* TSPCs showed a relatively higher proportion of FMOD^+^ cells (~54% in* LysM-Cre; Ampkα1^fl/fl^* group; ~38% in* Ampkα1^fl/fl^* group) than the NC group (~39% in* LysM-Cre; Ampkα1^fl/fl^* group; ~24% in* Ampkα1^fl/fl^* group). These results also implied that the knockdown of *Fbp2* could improve the tenogenic differentiation of TSPCs *in vivo* (Fig. 7D, E). Additionally, a greater percentage of SCX^+^Ki67^+^cells was observed in the regenerated tendon tissue of this group (Fig. 7F, G), which also confirmed that TSPCs after knocking down *Fbp2* have superior *in vivo* tenogenesis capacity.

Next, to further validate the role of FBP2 in tendon regeneration, an AAV vector carrying *Fbp2*-shRNA was injected into the tendon tissue of *LysM-Cre; Ampkα1^fl/fl^* mice and *Ampkα1^fl/fl^* mice after injury, while an AAV carrying an empty vector served as the control. RT-qPCR results suggested that *Fbp2*-shRNA decreased cellular *Fbp2* expression notably (Fig. S11A). Furthermore, gross observations revealed that both *LysM-Cre; Ampkα1^fl/fl^* mice and *Ampkα1^fl/fl^* mice transfected with *Fbp2*-shRNA retained the structural contour of tendons. In contrast, shRNA controls exhibited irregular and shrunken tendon surfaces at 4 and 8 weeks postoperatively (Fig. 7H). HE and Masson's trichrome staining revealed severe collagen disorganization in the tendons of the control groups. In contrast, tendons from the shRNA-*Fbp2* groups exhibited more aligned and thicker collagen fibers. The results suggest that local injection of *AAV*-*Fbp2-shRNA* effectively promoted tendon healing and improved healing quality at 4 and 8 weeks after the operation (Fig. 7I and Fig. S11B).

## Discussion

While AMPK has been implicated in tissue regeneration 19, 20, 37, the specific role of macrophage-derived AMPKα1 in tendon repair remains largely unexplored. In this study, we observed differential macrophage response between neonatal and adult tendons following injury and utilized transgenic mice to validate the impact of macrophage AMPKα1 activation on tendon regeneration. Additionally, transcriptomic sequencing analysis and *in vitro* experiments further elucidated the impact of macrophage AMPKα1 knockout on the biological functions of TSPCs and identified a novel regulating mechanism of the TNF-α/AKT/FBP2 axis in TSPCs, providing a new avenue of therapy for tendon repair. These findings provide potential regulatory targets and new insights into the regulation of TSPC function, as well as tendon tissue regeneration and repair.

It is well known that many human tissues and organs, such as the myocardium and spinal cord, exhibit strong regenerative capacity during the neonatal period, which declines markedly with age 4, 38. *Aurora* et al. observed different subtypes of macrophages in neonatal mice after myocardial infarction; however, the differences between neonatal and adult macrophages, as well as the mechanisms regulating these differences, remain to be determined 39. Our data suggest that AMPKα1 participates in regulating the differential changes in the function and phenotype of neonatal and adult macrophages and further elucidate the substantial role of macrophage AMPKα1 in tendon repair. Here, we used LysM-Cre transgenic mice to generate mice deficient in AMPKα1 in BMDMs. However, monocyte-derived macrophages do not necessarily represent all types of macrophages *in vivo*, and the LysM-Cre system deletes AMPKα1 from all myeloid cells—not only macrophages but also monocytes and granulocytes. In addition, during the process of injury, various components within the tendon tissue, including endothelial cells, TSPCs, other immune cells, and even local macrophages from neighboring tissues, such as calf muscle, participate in the entirety of injury regeneration and repair. While previous studies have shown that macrophage polarization influences tendon regeneration via soluble factors, other signaling pathways including STAT3, NF-κB and WNT, and extracellular vesicles, our findings reveal that AMPKα1 deficiency in macrophages not only promotes a pro-inflammatory phenotype but also disrupts TNF-FBP2 signaling in TSPCs. This non-enzymatic function of FBP2 links inflammatory signaling to mitochondrial metabolism, providing a mechanistic bridge between immune regulation and tendon repair.

Moreover, we identified *Fbp2* as a novel candidate gene in TSPCs that responds to macrophage-derived inflammatory signals, such as TNF-α, and mediates TSPC dysfunction. Recently, FBP2 has emerged as a prominent target enzyme in energy metabolism 23, 40, 41. Beyond its role as a rate-limiting enzyme in gluconeogenesis involved in glucose metabolism, FBP2 also has various functions, such as participation in mitochondrial biogenesis, cell cycle regulation, and transcriptional activation of hypoxia-inducible factors, among other biological processes 42, 43. In our study, we found no obvious changes at the gene level in FBP1, an isozyme of FBP2. We speculate that this could be attributed to the different distributions of the two isozymes: FBP1 is primarily detected in the liver and kidney, whereas FBP2 expression is more ubiquitous in tissues 44. Additionally, emerging evidence suggests that FBP2 can undergo conformational changes and regulate mitochondrial function via protein-protein interactions independent of its catalytic activity 36. While our data collectively support a non-enzymatic, regulatory role for FBP2 in mitochondrial homeostasis, we acknowledge that the precise molecular mechanism, particularly its potential non-enzymatic interactions, has not been fully elucidated. Moreover, future studies using stable genetic models or conditional *Fbp2* knockout strategies will be necessary to fully evaluate the long-term and inflammation-resistant effects of FBP2 depletion in TSPCs.

Mitochondria regulate a variety of cellular processes that are closely associated with tissue injury and repair, such as cell death, proliferation and differentiation, metabolic adaptation, and inflammation 45, 46. After tissue injury, excessive mitochondrial fission mediated by DRP1 mitochondrial translocation can lead to mitochondrial dysfunction 47, resulting in reduced ATP synthesis, massive ROS accumulation, reduced mitochondrial membrane potential, and ultimately leading to TSPC dysfunction 48, 49. Here, we demonstrated that TSPCs isolated from *LysM-Cre; Ampkα1^fl/fl^* mice displayed high expression of ROS and mitochondrial dysfunction, mainly caused by macrophage AMPKα1 depletion-induced immune imbalance. Notably, suppression of DRP1 hyperactivation attenuated these abnormal events in *Fbp2* knockdown experiments. Mitochondrial biogenesis is a genetically regulated program for mitochondrial proliferation during cell division and cellular stress, and stimulation of mitochondrial biogenesis has been demonstrated to facilitate tissue recovery after injury 50, 51. We also reported that *Fbp2* knockdown successfully restored the mitochondrial biogenic function by increasing the expression of PGC-1α and NRF1 proteins. PGC-1α is the master regulator of mitochondrial biogenesis that regulates metabolic rewiring in the face of different environmental cues and metabolic demand 52. Among these, PGC-1α-regulated targets related to mitochondrial biogenesis include the NRF1 and NRF2 and mitochondrial transcription factor A 46. Hence, activation of PGC-1α can promote the expression of mitochondrial biogenesis, increase mitochondrial mass, and reduce mitochondrial ROS levels. Moreover, one limitation of the present study is the lack of functional assessments. Beyond structural evaluation, functional indices including mechanical testing and gait analysis will be considered in future work to further validate tendon healing outcomes.

Overall, this study demonstrates that specific knockout of macrophage *Ampkα1* restricts the transition of the macrophage anti-inflammatory phenotype during tendon regeneration. Furthermore, it affects the mitochondrial function of TSPCs by regulating the key glycolytic enzyme, FBP2, thereby reducing cell stemness and tendon differentiation potential. In summary, the research elucidates the mechanism by which macrophage AMPKα1 influences tendon regeneration, identifying and deciphering novel targets for regulating stem cell and tendon regeneration capacity.

## Supplementary Material

Supplementary methods, figures and tables.
