# Supplementary material for: AMPKα1 Deficiency in Macrophages Impairs Tendon Regeneration and Tendon Stem Cell Function via TNF-α-FBP2 Signaling
Source: Int J Biol Sci. 2026 Jan 22;22(4):2047–64. doi: 10.7150/ijbs.125245 (PMC12905647; doi:10.7150/ijbs.125245)
Supplement: Supplementary file 1 — Supplementary methods, figures and tables. [file ijbsv22p2047s1.pdf]

Supporting materials

AMPKα1 Deficiency in Macrophages Impairs Tendon Regeneration and Tendon Stem Cell Function via a TNF-α – FBP2 Signaling

Methods

In vivo subcutaneously transplantation

To evaluate the effect of *Fbp2* knockdown on the tendon regeneration, cells were first treated with siRNA against *Fbp2* or negative control siRNA for 24 hours. Then, TSPCs (1×10^6 cells) with treatment were seeded on sterilized scaffolds, and implanted subcutaneously in the dorsum of athymic mice (6 – 8 weeks, BLAB/c nude mice) under anesthesia. Implants from 8 weeks were retrieved and fixed with 4% paraformaldehyde. Paraffin sections at a thickness of 4 μm were harvested, followed by HE, Masson's trichrome, and immunofluorescence staining.

Construction of a biomimetic parallel scaffold

Building upon our previously established protocol for collagen self-assembly, the biomimetic parallel scaffold was achieved through the following adapted procedure: A solution of Type I tropocollagen (Corning, 3,88 mg/mL) was injected into a dialysis bag (3500 Da), submerged in a phosphate buffer solution. Simultaneously, axial tensile force was applied to both ends of the dialysis bag using mechanical apparatus, and assembly was conducted at 37°C for 24 hours under constant temperature conditions. The scaffolds underwent overnight freeze-drying after freezing and were subsequently cross-linked for 4 hours under light-avoiding conditions using a cross-linking agent containing 1 wt% 1-ethyl-3-(3-dimethylaminopropyl)-carbodiimide in 80% ethanol. Finally, they underwent three alternating washes with distilled water and a 1% glycine solution, followed by freeze-drying and lyophilization for further use.

Flow cytometry

The mice were euthanized, and the harvested tendon was minced and digested completely with 3 mg/mL collagenase type I (Thermo Fisher Scientific) and 4 mg/mL dispase (Roche) at 37 °C for 1 h. After passing through a 70 μm strainer, the single-cell suspensions were incubated with the appropriate cocktail of antibodies (CD45, CD11b, F4/80, CD206, 1 μL/test) for 30 min at 4°C. Then, cells were washed two times with PBS and resuspended in 500 μL of flow buffer for analysis.

Macrophage phagocytosis

Efferocytosis was assessed in macrophages with autologous apoptotic TSPCs and evaluated through immunofluorescence staining. For the in vitro macrophage phagocytosis assays using fluorescence microscopy, apoptotic TSPCs were pre-labeled with PKH26. Subsequently, BMDMs were co-cultured with apoptotic TSPCs at the ratio of 1:10 of BMDMs to apoptotic TSPCs for 1 hour. Macrophages were then collected and washed three times with PBS, and fixed with 4% paraformaldehyde for 15 min to evaluate efferocytosis by microscopy.

Enzyme-linked immunosorbent assay (ELISA)

To measure IL-6, TNF-α, IL-4 and IL-10 levels in culture supernatants, BMDM supernatants were collected, centrifuged at 500 × g for 5 min to remove cell debris and then assayed using the IL-6, TNF-α, IL-4 and IL-10 Mouse commercial ELISA Kit (ExCell Bio, Inc., Jiangsu, China) according to the manufacturer's instructions.

CFU-F assays

For CFU-F assay, single-cell suspensions of TSPCs (1000 cells/well) for 12 – 14 days in growth medium and fixed with 4% paraformaldehyde. Then, 0.1% crystal violet solution (Solarbio, Beijing, China) was used to stain the cells. Colonies of >50 cells were defined as single colony unit, and the number of clusters was counted using Image J 1.52a software.

Scratch assay

A wound healing assay was also employed to assess the migration of TSPCs. TSPCs were seeded in 6-well plates and cultured until confluent. A straight line was scratched using a pipettor spear head in each well. The 6-well plate was washed twice with PBS buffer to remove floating cells. At 0, 24 and 36 hours, scratch wound healing was recorded using an inverted microscope, and the scratch width was measured by Image J software for statistical analysis.

Macroscopic evaluation of regenerated tendons.

At 1- and 4-week postoperatively, mice were euthanized and the Achilles tendon was fully exposed. Then the full-length tendon complexes including partial gastrocnemius and calcaneus were harvested, and photographed using a camera (Nikon, Japan).

ROS assay

Mitochondrial ROS were measured using Mito SOX following the manufacturer's instructions. First, the cells were washed with PBS, then cells were incubated in 5 μM Mito SOX Red solution (Yeasen, China) at 37 °C for 10 min. Finally, the red fluorescence of cells was calculated by a flow cytometer (BD Accuri C6).

Cellular reactive oxygen species were tested by using DCFH-DA. A final working concentration of 10 μM DCFH-DA was added and incubated at 37 °C for 20 min. Then the cells were washed by prewarmed PBS. Finally, the cells were checked with a laser-scanning microscope (LSM 510, Zeiss, Germany).

Assessment of F6P

The content of F6P was measured by using the fructose-6-phosphate assay kit (Beyotime, China) by examining the fluorescence intensity of Ex/Em (535/587 nm).

Supplementary Figures

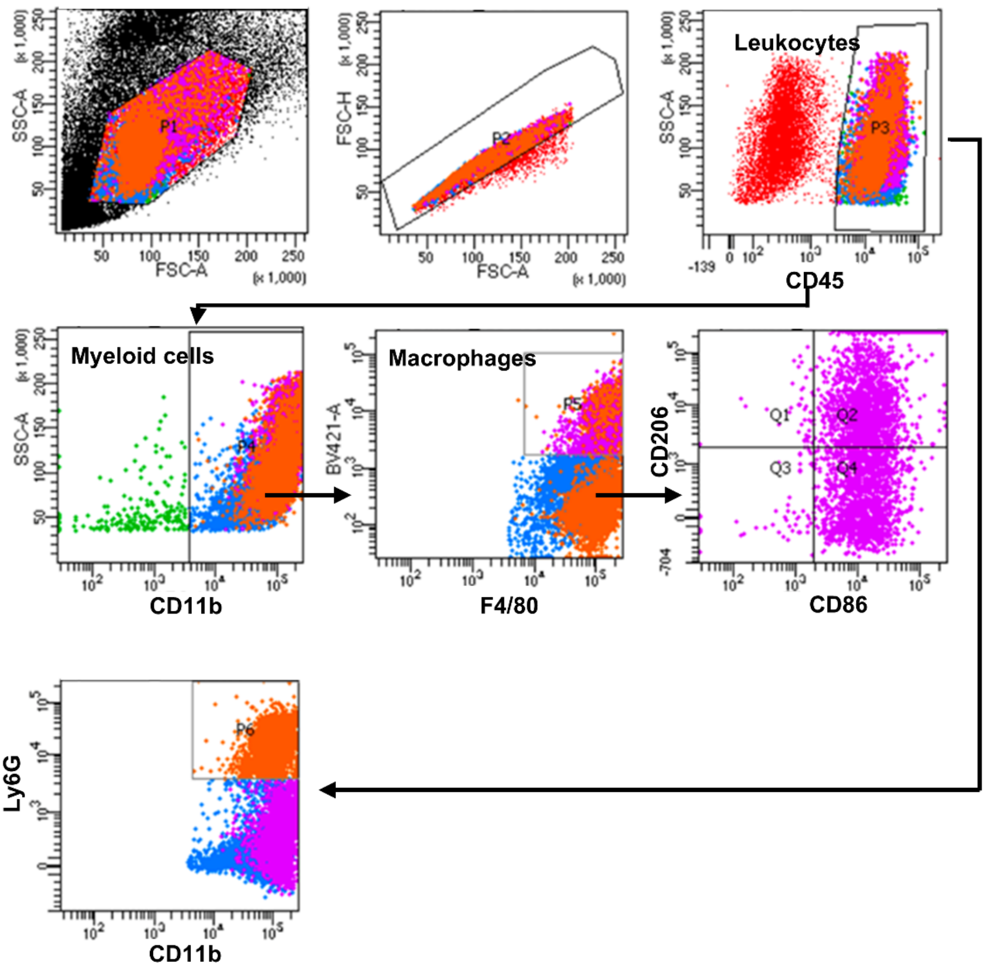

Fig. S1 Gating strategy for flow cytometric analysis.

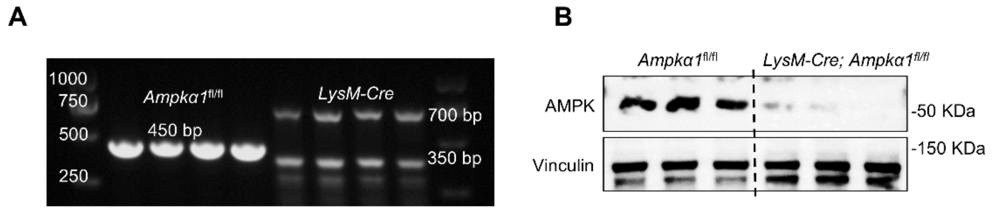

Fig. S2 Validation and characterization of *LysM-Cre; Ampkα1$^{fl/fl}$* mice. **(A)** Agarose gel electrophoresis qRT-PCR products of *LysM-Cre; Ampkα1$^{fl/fl}$* mice. **(B)** Western blotting analysis of AMPKα1 expression in bone marrow-derived macrophages (BMDMs) of control and *LysM-Cre; Ampkα1$^{fl/fl}$* mice.

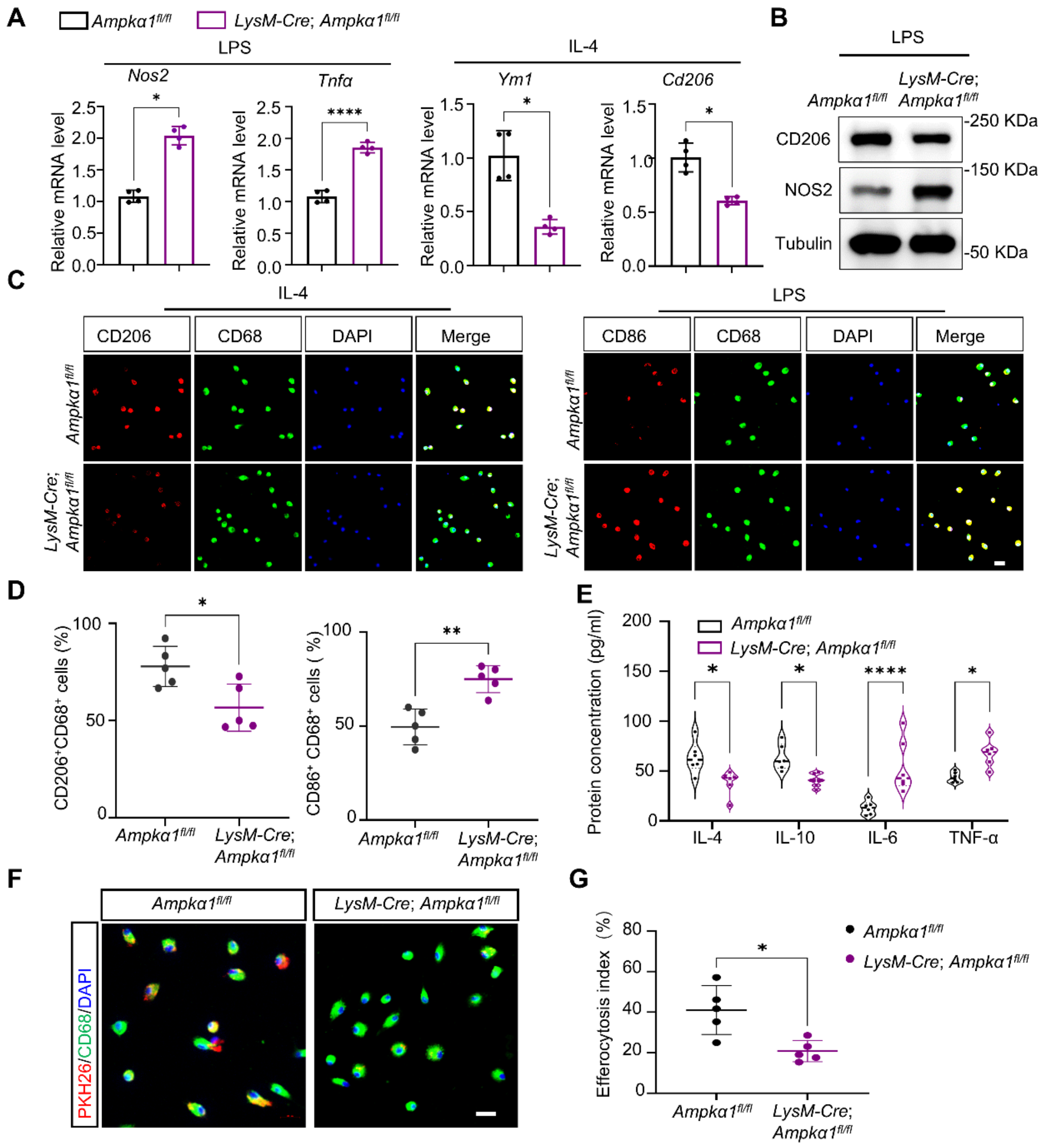

Fig. S3 Knocking out *Ampkα1* hinders macrophage transition to an anti-inflammatory phenotype and its phagocytic function. **(A)** The expression of *Nos2*, *Tnf-α*, *Ym1* and *Cd206* in the polarization of macrophages under different treatments by qRT-PCR analysis (n = 4). **(B)** Western blotting analysis of CD206 and NOS expression in the macrophages under lipopolysaccharide (LPS) treatments. **(C)** Immunofluorescent co-staining of CD206 or CD86 (red) with CD68 (green) in the polarization of macrophages under different treatments. Scale bars: 20 µm. **(D)** Left: proportion of CD206 and CD68 double-positive cells among CD68-positive cells (n = 5). Right: proportion of NOS2

and CD68 double-positive cells among CD68-positive cells (n = 5). **(E)** Expression levels of the cytokines IL-4, IL-10, IL-6, and TNF-α in macrophage supernatants of control and *LysM-Cre; Ampkα1*$^{fl/fl}$ group were detected by ELISA. **(F)** Immunofluorescent co-staining of PKH26 with CD68 in the macrophages (n = 6). Red: PKH26 labeled apoptotic TSPCs; Green: CD68 labeled BMDMs. Scale bars: 20 μm. **(G)** Percent efferocytosis was quantified as the number of macrophages with engulfed apoptotic cells as a percentage of total macrophages (n = 5). * $p < 0.05$, ** $p < 0.01$, and **** $p < 0.0001$.

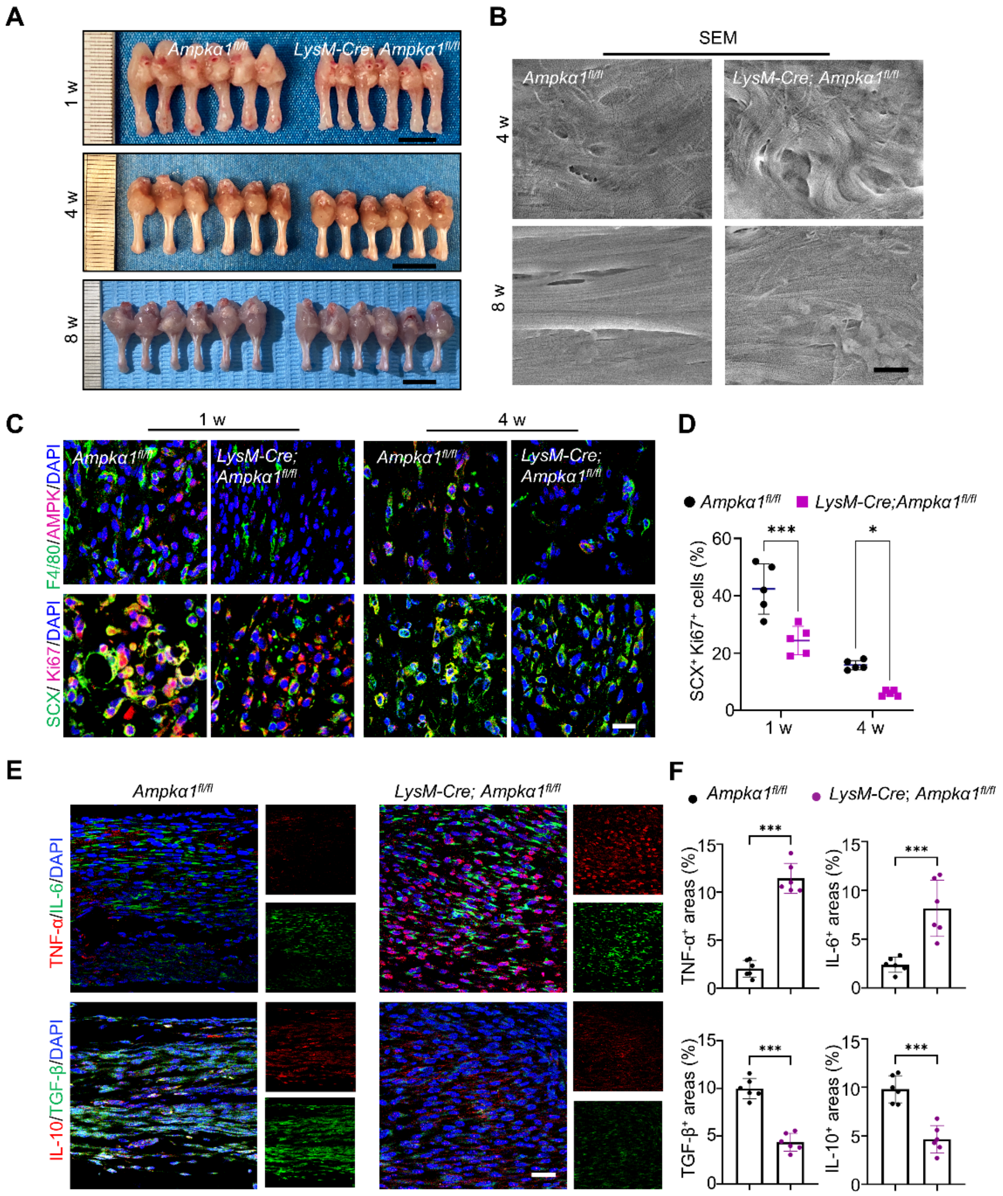

Fig. S4 Macrophage AMPKα1 deficiency impairs tendon regeneration and repair capacity. **(A)** Gross view of tendons in newborn and adult mice at 1-, 4- and 8-week after injury. Scale bars: 1 cm. **(B)** Representative scanning Electron Microscope (SEM) images illustrating the microstructural characteristic of regenerated collagen fibers in the control and *LysM-Cre; Ampkα1*$^{fl/fl}$ group at 4- and 8-week after injury. Scale bars: 1 μm. **(C)** Immunofluorescent co-staining of AMPK (red) with F4/80 (green), Ki67 (red) with SCX (green) in the control and *LysM-Cre; Ampkα1*$^{fl/fl}$ group at 1- and 4-week after injury. Scale bars: 20 μm. **(D)** Proportion of SCX and Ki67 double-positive cells

among SCX-positive cells (*n* = 5). **(E)** Immunofluorescence staining results showed the protein expression levels of TGF-β, IL-10, IL-6, and TNF-α of *LysM-Cre; Ampkα1*$^{fl/fl}$ group and control group. Scale bars: 50 μm. **(F)** Quantification analysis of p-AKT^{+}SCX^{+}-positive cells and p-AKT^{+}CD146^{+}-positive cells (*n* = 6). * $p < 0.05$, and ***$p < 0.001$.

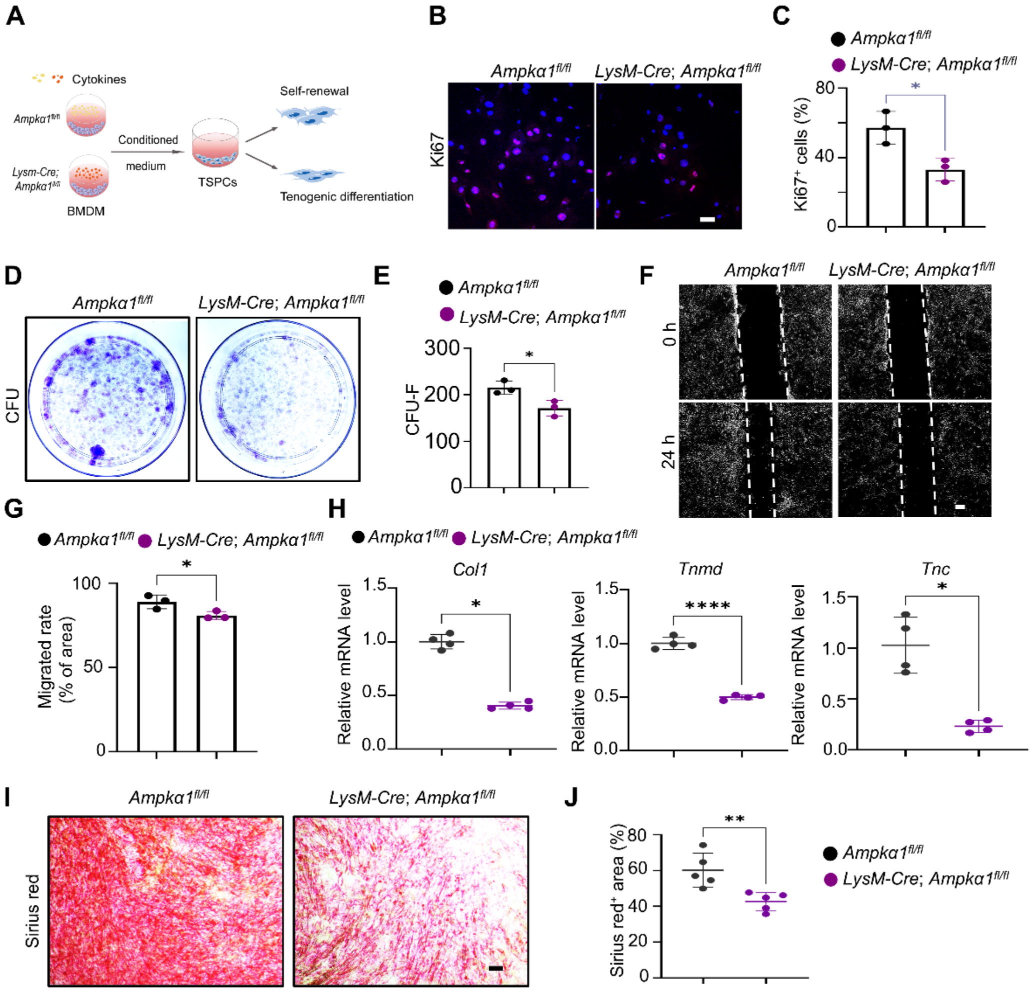

Fig. S5 Macrophage knockout of *Ampka1* impairs the proliferation, migration, and differentiation of TSPCs. **(A)** Schematic representation of TSPCs stimulated by supernatant of *LysM-Cre; Ampka1$^{fl/fl}$* and control mouse-derived macrophages. **(B)** The protein level of Ki67 in TSPCs stimulated with different culture supernatants was analyzed by immunofluorescent staining. Scale bars: 20 µm. **(C)** Quantitative analysis of Ki67-positive cells (*n* = 3). **(D)** Photograph of crystal violet staining in both TSPCs groups after 14 days of culture. **(E)** Quantification of crystal violet staining (*n* = 3). **(F)** Images of cell scratch assay for migration of TSPCs cultured for 0 and 24 h in both supernatant groups. Scale bars: 200 µm. **(G)** The percentage of recovered area in scratch assay (*n* = 3). **(H)** The expression of *Col1*, *Tnmd*, and *Tnc* in both TSPCs groups was evaluated by qRT-PCR analysis (*n* = 4). **(I)** Photomicrographs of Sirius red staining of

both TSPCs groups were cultured for two weeks after tenogenic differentiation induction. Scale bars: 100 μm. **(J)** Quantification of Sirius red-positive areas ($n = 5$). *$p < 0.05$, **$p < 0.01$, and ****$p < 0.0001$.

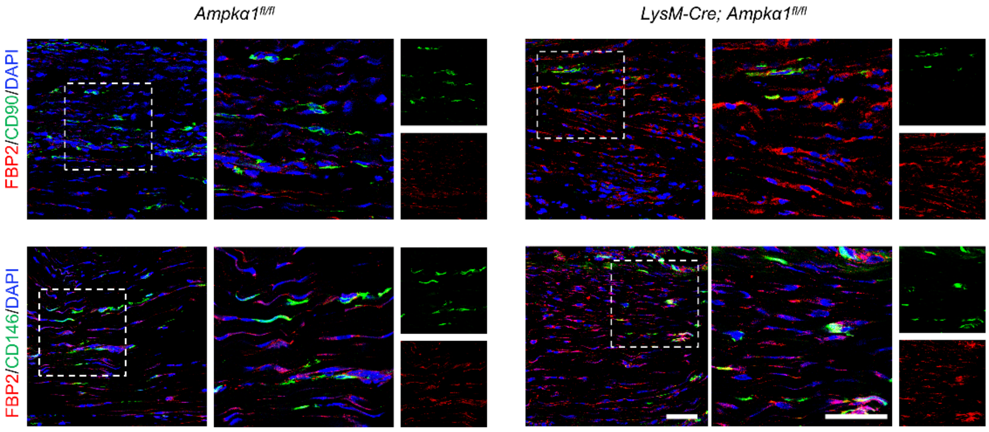

Fig. S6 The colocalization of FBP2 and tendon-specific markers (CD146 and CD90) in tendon tissue. Scale bars: 50 μm.

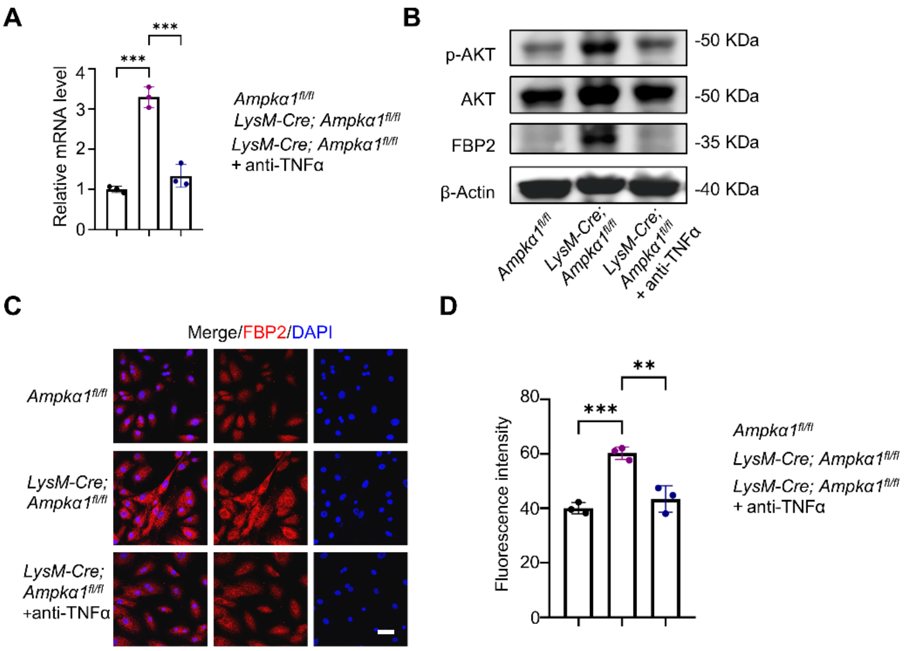

Fig. S7 TNF-α increased FBP2 levels through PI3K /AKT signaling. **(A)** The expression of *Fbp2* in TSPCs treated with a TNF-α neutralizing antibody was evaluated by RT-PCR analysis (n = 3). **(B)** Western blotting analysis of p-AKT, AKT and FBP2 expression in TSPCs treated with a TNF-α neutralizing antibody. **(C)** Immunofluorescence staining results showed the protein expression levels of FBP2 (red) in TSPCs treated with a TNF-α neutralizing antibody. **(D)** Quantification of FBP2 fluorescent intensity (n = 3). Scale bars:50 μm. **$p < 0.01$, and ****$p < 0.0001$.

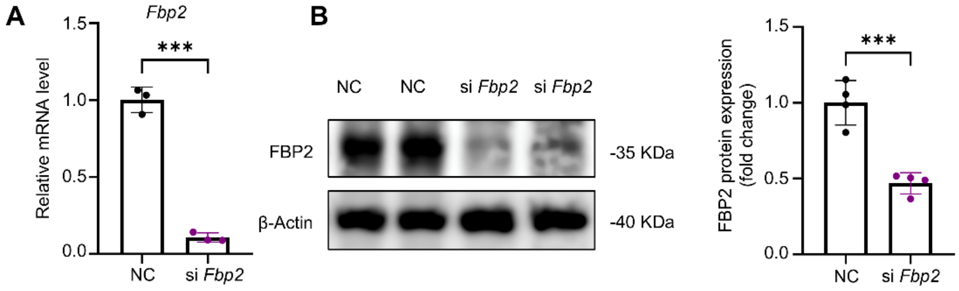

Fig. S8 Verification of the FBP2 knockdown efficiency. (**A)** The expression of *Fbp2* after siRNA treatment was evaluated by qRT-PCR analysis (n = 3). (**B)** Western blotting analysis of FBP2 expression after siRNA treatment (n = 4). ***p < 0.001.

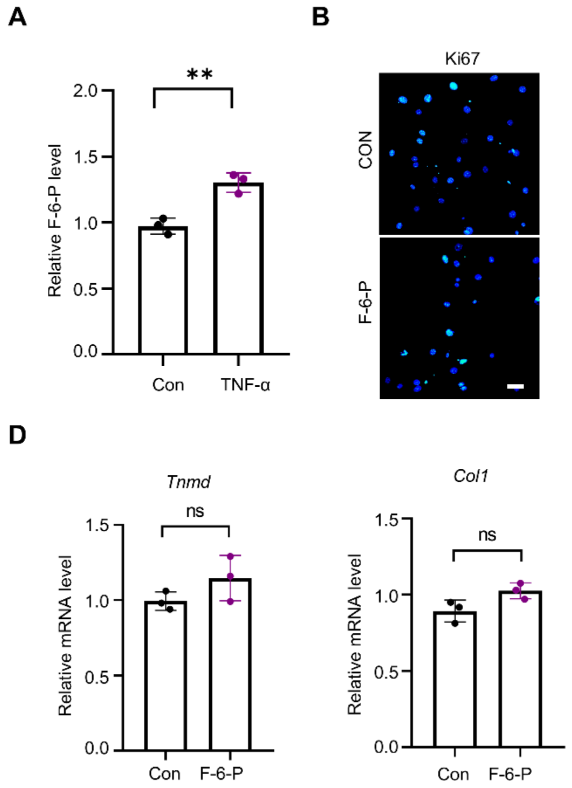

Fig. S9 Assessment of F6P content and its impact on TSPCs. **(A)** The levels of F6P of TSPCs stimulated by TNF-α was measured by using the fructose-6-phosphate assay kit. **(B)** Immunofluorescence staining results showed the protein expression levels of Ki67 in TSPCs were treated with F-6-P (10mM) for 24 h, compared to control group. Scale bars: 20 µm. **(C)** Quantification of ratio of Ki67 positive cells (n = 3). **(D)** The expression of *Tnmd*, and *Col1* in TSPCs treated with F-6-P for 24 h was evaluated by RT-PCR analysis (n = 3). ns: no significance; **$p < 0.01$.

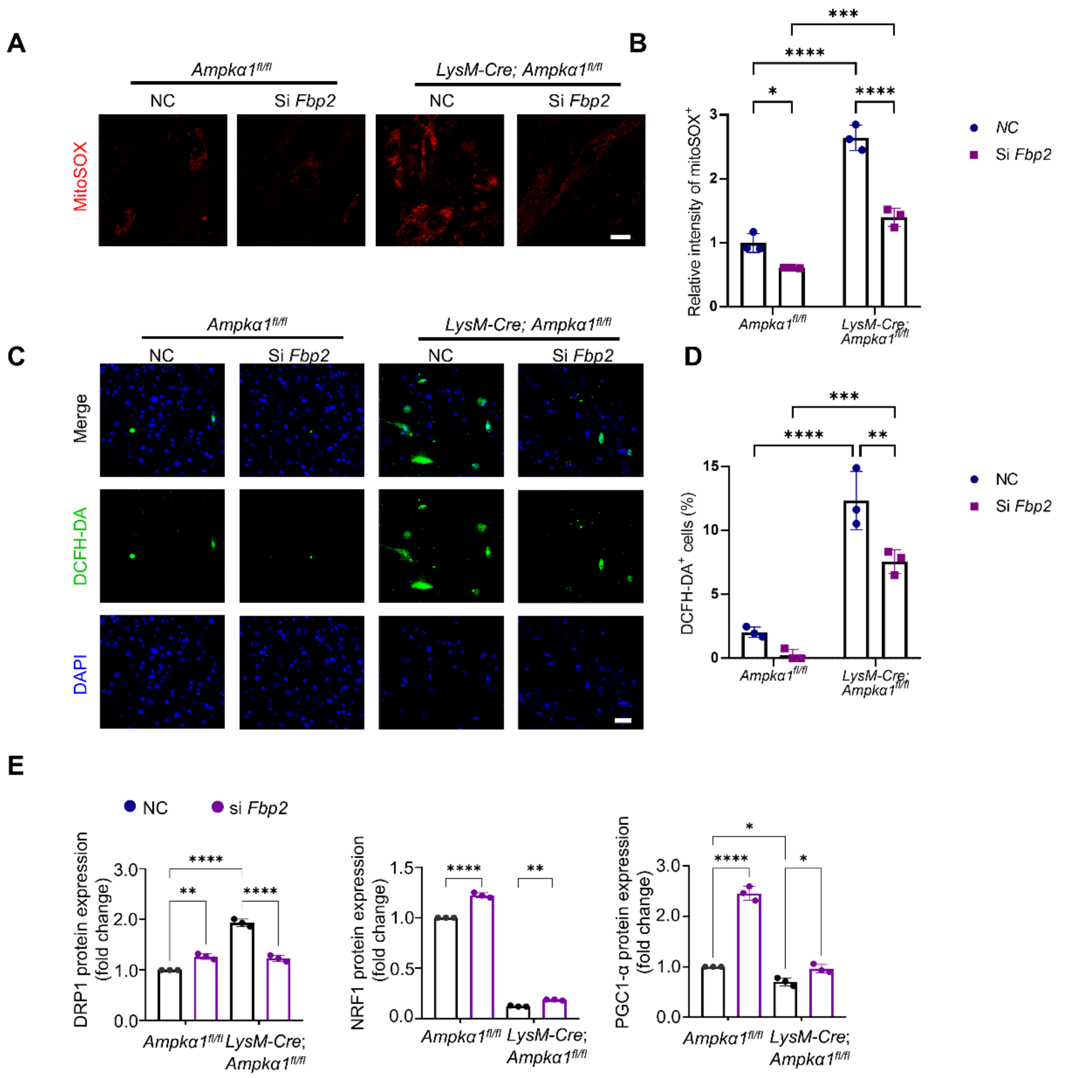

Fig. S10 The impact of *Fbp2* knockdown on TSPCs mitochondria. (A) Immunofluorescence staining results showed the fluorescence intensity of mitoSOX in *Fbp2*-knockdown TSPCs, compared to NC group. Scale bars: 20 µm. (B) Quantification of mitoSOX fluorescent intensity (n = 3). (C) Immunofluorescence staining results showed the fluorescence intensity of DCFDA in *Fbp*2-knockdown TSPCs, compared to NC group. Scale bars: 50 µm. (D) Quantification of DCFDA fluorescent intensity (n = 3). (E) Quantification of western blotting results in Figure 6J (n = 3). *$p < 0.05$, **$p < 0.01$, ***$p < 0.001$ and ****$p < 0.0001$.

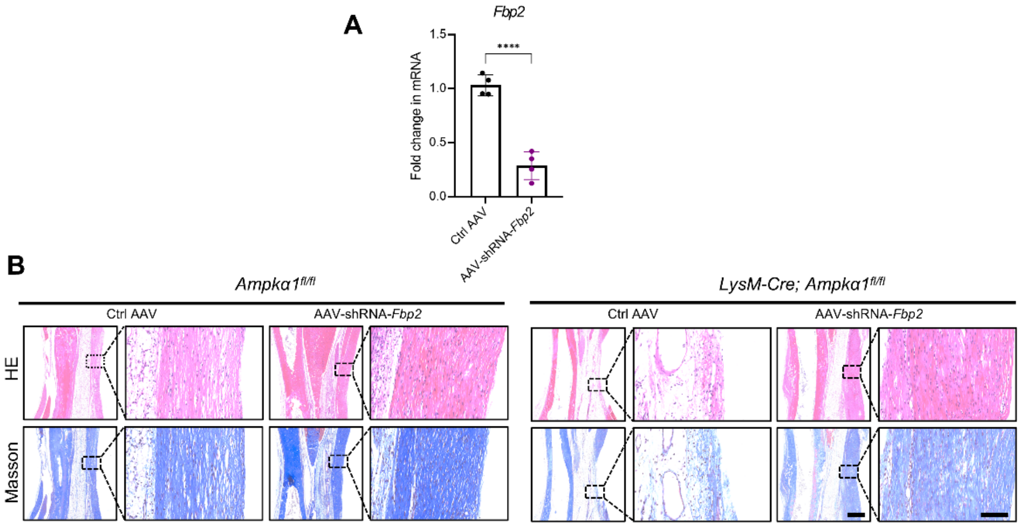

Fig. S11 *Fbp2* knockdown in TSPCs notably enhances tendon regeneration. **(A)** The knockdown efficiency of shRNA targeting Fbp2 in TSPCs were examined with qRT-PCR (n = 3). **(B)** Representative HE staining and Masson trichrome staining of the tendon from *LysM-Cre; Ampkα1$^{fl/fl}$* mice and *Ampkα1$^{fl/fl}$* mice injected with *AAV-Fbp2-shRNA* and control *AAV-shRNA* at 4 weeks after injury. Low magnification image scale bars: 500 µm; high magnification image scale bars: 100 µm.**** $p < 0.0001$.

Supplementary Tables

Table S1. The primers used for qRT-PCR identification.

Gene	Primer sequences（5’-3’）
Ampkα1$^{flox/flox}$ Forward	CCCACCATCACTCCATCTCT
Ampkα1$^{flox/flox}$ Reverse	AGCCTGCTTGGCACACTTAT
LysM-Cre Mutant	CCCAGAAATGCCAGATTACG
LysM-Cre Common	CTTGGGCTGCCAGAATTTCTC
LysM-Cre Wild type	TTACAGTCGGCCAGGCTGAC

Table S2. The commercial reagents.

Reagents	Supplier
Formaldehyde Fixative Solution	Servicebio, China
Glutaraldehyde	Sigma, USA
Decalcification Solution	Servicebio, China
0.1% Triton Solution	Solarbio, China
5% Goat Blocking Serum	Zsbio, China
Bovine Serum Albumin	Sigma, USA
Hematoxylin-Eosin Staining Kit	Solarbio, China
MASSON Staining Kit	Beijing Solarbio
Picrosirius Red Staining Kit	Solarbio, China
Low-glucose DMEM	Hyclone, USA
Fetal Bovine Serum	Gibco, USA
Penicillin/streptomycin	Hyclone, USA
L-glutamine	Hyclone, USA
Trypsin	Hyclone, USA
Dimethyl Sulfoxide (DMSO)	Solarbio, China
Recombinant TGF-β Factor	Peprotech, USA
GDF-5 Factor	R&D Systems, USA
Recombinant Mouse M-CSF Protein	Novoprotein, China
Recombinant Mouse TNF-α Protein	Novoprotein, China
lipopolysaccharide	Sigma, USA
Recombinant Mouse IL-4 Protein	Peprotech, USA
Collagenase I	Solarbio, China
Dispase	Roche, Switzerland
Lymphoprep™	Stemcell Technologies, Canada
0.1% Crystal Violet Staining Kit	Solarbio, China
Cell Transfection Kit	Polyplus, France

Reagents	Supplier
PKH26 Red Staining Kit	Solarbio, China
Staurosporine	MedChemExpress, USA
Mouse ELISA Kit	MeiMian, China
Trizol	Thermo Fisher Scientific, USA
DEPC Water	Beyotime, China
Tris-HCl	Beyotime, China
Reverse Transcription Kit	Takara, Japan
SYBR Green Real-time Quantitative PCR Kit	Roche, Switzerland
RIPA Lysis Buffer	Thermo Fisher Scientific, USA
Protease/Phosphatase Inhibitor Cocktail	Thermo Fisher Scientific, USA
BCA Protein Quantification Kit	Thermo Fisher Scientific, USA
High-purity Heat-resistant DNA Polymerase	Solarbio, China
Tricolor Pre-stained Protein Standard	Biotides, China
Mouse Tail Collagen Solution	Corning, USA
1-(3-Dimethylaminopropyl)-3-ethylcarbodiimide	Tcichemicals, China
N-Hydroxysuccinimide	Sigma, USA
JC1 staining	Solarbio, China
Mito SOX Red	Yeasen, China
MitoTracker Red	Beyotime, China
HE staning	Solarbio, China
Sirius Red staining	Solarbio, China
Masson trichrome staining	Solarbio, China

169

Table S3. The target sequences for *Fbp2* knockdown and negative control.

Gene	Primer sequences（5'-3'）
si *Fbp2* Forward	GCUAUUUACAGAAAGACCATT
si *Fbp2* Reverse	UGGUCUUUCUGUAAAUAGCTT
si NC Forward	UUCUCCGAACGUGUCACGUTT
si NC Reverse	ACGUGACACGUUCGGAGAATT

Table S4. List of RT-qPCR primers.

Gene	Primer sequences（5’-3’）
Actin-F	CATGTTTGAGACCTTCAACACCC
Actin-R	TGGCATAGAGGTCTTTACGGATG
Gapdh-F	CATGGCCTTCCGTGTTCCTA
Gapdh-R	CCTGCTTCACCACCTTCTTGA
Ym1-F	CCAGTTGGGCTAAGGACAGG
Ym1-R	CAGGTGAGTACACAGGCAGG
Cd206-F	GTGGAGTGATGGAACCCCAG
Cd206-R	CTGTCCGCCCAGTATCCATC
Tnf-F	CCTGTAGCCCACGTCGTAGC
Tnf-R	AGCAATGACTCCAAAGTAGACC
Nos2-F	GTTCTCAGCCCAACAATACAAGA
Nos2-R	GTGGACGGGTCGATGTCAC
Col1-F	CCACCCCAGCCGCAAAGAGTC
Col1-R	GTCATCGCACACAGCCGTGC
Tnmd-F	GGGCTGTCACATTCTAAATGCAG
Tnmd-R	TTCTTCTTCTCGCCGTTGCT
Tnc-F	TTCTTCTTCTCGCCGTTGCT
Tnc-R	AGAGGGTATGCTATAAGCCAGAA
G6pc-F	TCCGTGCCTATAATAAAGCAGT
G6pc-R	TGGCTTTTTCTTTCCTCGAAAG
Pgam1-F	CAGGTAAAGATCTGGAGACGAT

Pgam1-R	CTTGCTGATGTTGCTGTAGAAG
Eno1-F	TTCGCACCTAACATCCTGGAGAAC
Eno1-R	GAACTCGGAGGCAGCCACATC
Pfk1-F	ACGGTATACATCGTGCATGAT
Pfk1-R	GATGTTGTAGGTGCGGAGATTC
Fbp2-F	ACAGGACAAGGAGTAGATCTCT
Fbp2-R	TAGCAGCATCAAAATACTTGGC
Gpi1-F	ATTGCTCTGCATGTAGGTTTTG
Gpi1-R	CTTGGTGATGTACTTTCCGTTG
Pgk1-F	CAAATTCTGCTTGGACAATGGA
Pgk1-R	CCACACAATCCTTCAAGAACAG
Pkm-F	TATCATTGCCGTGACTCGAAAT
Pkm-R	AAGTTTACACGAAGGTCGACAT
Aldoc-F	ATGGCGCTGTGTACTAAAAATC
Aldoc-R	TGTAACATACTGGCAACGTTTG
Hk1-F	ATTAAGAAGCGAGGGGACTATG
Hk1-R	CTCCCCATTCCGTGTTAATACA
Slc2a3-F	TGGGATCAATGCTGTGTTCTAT
Slc2a3-R	CCAGGAACAGAGAAACTACAGT
mt DNA	GCCAGCCTGACCCATAGCCATAAT
mt DNA	GCCGGCTGCGTATTCTACGTTA
nu-DNA	TTGAGACTGTGATTGGCAATGCCT
nu-DNA	CCAGAAATGCTGGGCGTACT

Table S5. The list of antibodies.

Antibody	Catalog No.	Supplier
NOS2	18985-1-AP	Proteintech, USA
CD206	18704-1-AP	Proteintech, USA
F4/80	ab90247	Abcam, USA
CD86	13395-1-AP	Proteintech, USA
CD68	ab303565	Abcam, USA
p-AMPK	ab90247	Thermo Fisher Scientific, USA
AMPK	#2532	Cell Signaling Technology, USA
SCX	ab58655	Abcam, USA
Ki67	ab16667	Abcam, USA
TNC	MA5-16086	Thermo Fisher Scientific, USA
COL1	14695-1-AP	Proteintech, USA
FMOD	60108-1-Ig	Proteintech, USA
FBP2	PA5-97265	Thermo Fisher Scientific, USA
FBP2	sc-166097	Santa Cruz，USA
GAPDH	TA-08	Zsbio, China
Vinculin	66305-1-lg	Proteintech, USA
β-Tubulin	10068-1-AP	Proteintech, USA
ACTB	TA-09	Zsbio, China
DRP1	sc-101270	Santa Cruz，USA
PGC-1α	sc-518025	Santa Cruz，USA
NRF-1	sc-28379	Santa Cruz，USA
HRP-linked anti-mouse IgG	ZB-2305	Zsbio, China
HRP-linked anti-rabbit IgG	ZB-2301	Zsbio, China

**Antibody**	**Catalog No.**	**Supplier**
FITC-labeled goat anti-mouse IgG (H + L)	ZF-0312	Zsbio, China
FITC-labeled goat anti-rabbit IgG (H + L)	ZF-0311	Zsbio, China
FITC-labeled goat anti-rat IgG (H + L)	ZF-0315	Zsbio, China
Rhodamine labeled goat anti-mouse IgG (H + L)	ZF-0313	Zsbio, China
Rhodamine labeled goat anti-rabbit IgG (H + L)	ZF-0316	Zsbio, China
Rhodamine labeled goat anti-rat IgG (H + L)	ZF-0318	Zsbio, China
Mounting Medium with DAPI	ZLI-9557	Zsbio, China
